# Children’s social networks in developmental psychology: A network approach to capture and describe early social environments

**DOI:** 10.3389/fpsyg.2022.1009422

**Published:** 2022-10-14

**Authors:** Nicole Burke, Natalie Brezack, Amanda Woodward

**Affiliations:** ^1^Department of Psychology, New York University, New York, NY, United States; ^2^Department of Psychology, University of Chicago, Chicago, IL, United States

**Keywords:** social network analysis, social networks, social cognition, social relationships, cognitive development

## Abstract

Psychologists are interested in understanding how early social environments impact children’s behavior and cognition. Early social environments are comprised of social relationships; however, there have been relatively few tools available to quantify the depth and breadth of children’s social relationships. We harnessed the power of social networks to demonstrate that networks can be used to describe children’s early social environments. Descriptive data from American children aged 6 months–5 years (*n* = 280; 47% female, 56% White) demonstrates that network properties can be used to provide a quantitative analysis of children’s early social environments and highlights how these environments vary across development. Social network methodology will provide researchers with a comprehensive picture of children’s early social experiences and improve studies exploring individual differences.

## Introduction

Developmental psychologists who are interested children’s early social cognition have a keen interest in understanding how children’s early social experiences shape their behavior, thoughts, and mind. Children are born into a world that is rich with social information – they are a part of varied social groups and cultures and children must learn to navigate these social organizations with different rules and customs. While children certainly learn about the world through their own action (Piaget and Inhelder, 1969), children come to learn about the social world through their social relationships; they gain social cognitive capacities by interacting with their social relationships and they learn about social conventions and rules by interacting and observing their social relationships (Vygotsky, 1978; Gaskins and Paradise, 2010).

Sociocultural theories have been the leading theories to understand how social interactions affect the developing child – these theories emphasize the cultural context in which children learn and develop and how social interaction is the engine of learning and development (Vygotsky, 1962, 1978; Tomasello, 2001, 2009). The focus of these theories is how social interactions shape children’s knowledge construction and that social interactions take place in different cultural contexts. Sociocultural theories highlight how important social interactions are for development. In addition to the focus on the child as a social learner, these theories explore how children’s experience can vary across culture.

Sociocultural theories do an excellent job to highlight how variation at the level of culture impacts children’s learning; however, there is growing interest in the field to further explore how variations in children’s regular social contact and early social experience impact social cognitive development (see Fan et al., 2015 for an example). Interestingly, this dimension of early social experience – the people a child has regular social contact with – has largely been understudied in prior developmental work. A fundamental aspect of social experience is the day-to-day interactions that children have with other people. A number of studies have investigated aspects of these social relationships, for example the effects of contact with people from different racial groups on prejudice (Rutland et al., 2005; Weisman et al., 2015), the effects of multilingual social environments on social cognition and social learning (Byers-Heinlein and Werker, 2009; Barac and Bialystok, 2012; Howard et al., 2014; Yow and Markman, 2015), and the effects of siblings on social cognition (for examples see Perner et al., 1994; Jenkins and Astington, 1996; Kennedy et al., 2015).

In a typical week, young children interact with a range of social partners. Young children engage with their family members in the home, they see neighbors on the weekends, they visit their local community center, they go to library story hour and see the librarian and other kids, and they might attend daycare or preschool and interact with teachers and fellow classmates. Young children’s early social relationships provide data to them about how the social world is structured and how it functions. Observing and interacting with different social relationships likely affects the skills children come to develop in social interactions, as well as inform their early attitudes and thoughts about different social groups (Vygotsky, 1978; Gaskins and Paradise, 2010).

Although this dimension of early social experience provides rich data to children about the structure and function of their early social world, there are relatively few tools and frameworks available that can describe the breadth and depth of children’s early social relationships. We argue that social networks are a powerful tool and framework that developmental psychologists can use to inform our study of early social cognition. Psychologists often have research questions that ask about an individual’s attitudes, cognition, or behavior, but to understand an individual’s cognition or behavior, it is necessary to consider how the individual is embedded in a broader social context. Bronfenbrenner’s ecological systems theory is the leading developmental theory to explore how a child is embedded in a broader social context. He argues that children’s social environment can be thought about in terms of different layers – everything from the macrosystem that describes the broader culture that children live in to the microsystem, which describes the interpersonal relationships children have with family members and peers (Bronfenbrenner, 1977). We argue that a social network perspective provides an excellent methodological tool and perspective to complement this framework; social networks can consider a broad range of social partners, as well as provide metrics that allow researchers to be clear and specific about what aspects of the early social environment they are capturing and describing.

A social network perspective can inform the study of early social cognition in two important ways. First, social networks can capture and describe important aspects of children’s early social experience and provide a novel way to explore children’s complex and embedded early social environments. Second, a social network framework will generate questions and hypotheses not previously asked to better understand how early social experience affects social cognition.

### The complicated answer to “What is a social network?”

Before we outline the benefits to be gained by using a social network perspective, it is important to establish an operational definition of a social network. An operational definition of a social network is no small task because the term “social network” refers to several different literatures with several different meanings; social networks are a powerful, flexible tool that can be used to describe and study network structure across several different disciplines.

Simply put, a network is a set of objects or actors and the connections between them (Wasserman and Faust, 1994; Perry et al., 2018). Network science shares a theoretical focus on ties between objects; however, there is a wide breadth of questions that can be asked using a network perspective (Perry et al., 2018). A social network perspective can be used to study a variety of groups – adults, adolescents, animals – and it is used to ask several different kinds of research questions at multiple levels of analysis. A social network could detail the connections between individuals at the level of the social system; for example, social networks have been used to ask about how romantic relationships in a particular high school related to the spread of sexually transmitted diseases among students (Bearman et al., 2004). This kind of network can also be explored in animals; a study of endangered killer whales discovered that in years of high food availability, there was more interconnectedness in the social network of whales (Foster et al., 2012). These networks are called sociocentric networks or whole networks (Perry et al., 2018). A social network perspective could also be used to explore how population-level characteristics relate to individual behavior; for example, a study with cowhead birds showed that birds in dynamic social networks, where individuals were replaced over time, had more reproductive success than birds in a static social network (White et al., 2010; Gersick et al., 2012). A social network could also delineate the people emotionally close to or immediately surrounding an individual; these are called egocentric networks (Robins, 2015; Perry et al., 2018). A social network perspective can be used to ask how the personal social network of an individual affects their mental and physical health (Haines and Hurlbert, 1992; Smith and Christakis, 2008), whether the presence of a smoker in an adolescent’s peer network will influence whether they become a smoker (Alexander et al., 2001), if social network size is related to brain size in adults (Bickart et al., 2011), or even if the language diversity of adults’ social networks relate to their theory of mind skills (Navarro et al., 2022; Tiv et al., 2022).

These examples demonstrate that a social network perspective is powerful and flexible because it can be used to study social phenomenon at several different levels of analysis and across several different populations. Because a social network perspective can be used to study network structure across several different disciplines there has been an explosion of network research in the past several decades (Borgatti and Halgin, 2011). This explosion of research is seen within the psychological sciences as well, with substantial increases of network research in education (McPherson et al., 2001), social psychology (Clifton and Webster, 2017) and even in developmental psychology (Neal, 2020). Network theorists argue that we have seen this explosion of research because networks can be studied at multiple levels and a social network perspective can generate a lot of rich data – both qualitative and quantitative – that make it an excellent tool for studying social phenomenon.

Yet, despite this increase of network research in psychological sciences, and in developmental psychology in particular, very little work has explored the personal social networks of young children (Neal, 2020). Social networks have been used in developmental psychology for the past several decades, but they have been used either in adolescent samples or answer questions at the level of the social system (see Neal, 2020 for review). Developmentalists have used network methodology to study bullying in adolescent peer networks (Neal and Veenstra, 2021; Veenstra and Huitsing, 2021), how peer networks can influence children’s reading skills (Cooc and Kim, 2017) and early academic skills (Hanish et al., 2007) in the classroom, and they have even used networks to map and describe the racial composition of classrooms (Rodkin et al., 2007). If you put in the search term “social networks” on the APA PsycArticles database from 1990 to early 2022 and search for developmental samples (birth to 12-years-old) there are only 51 articles. Most of the 51 articles are looking at sociocentric social networks or networks that are bounded by the classroom or school. Young children’s egocentric social networks have largely been ignored by prior work – we know very little about the composition of these networks or how aspects of networks might influence social cognition.

This vacuum of research is striking given the longstanding interest in children’s early social context among developmental psychologists. It is important to understand the composition of social networks for infants and young children because a child’s social network captures most of their early social experience. Infants come to learn about the social world through their social relationships; they gain social cognitive capacities by interacting with their social relationships and they learn about social conventions and rules by interacting and observing their social relationships (Vygotsky, 1978; Gaskins and Paradise, 2010).

Although there is substantial interest in understanding how variation in early social environments impacts social cognitive development, there is no unified framework to think about how social experience might affect children’s social cognitive development. Prior developmental work has been limited in scope because it has only focused on single aspects of experience and how that relates to social cognition; for example, how does the number of siblings a child have relate to their theory of mind ability? When early social experience is only conceptualized as isolated components, it is impossible to consider how various aspects of early social experience relate to each other. As stated above, there is evidence to suggest that exposure to multiple languages is associated with gains in social cognitive abilities; however, it is possible that multilingual environments covary with other aspects of experience that might be important for social cognitive development, such as interacting with more people outside the immediate family or interacting with a larger number of people on a regular basis. Another limitation of prior developmental work is that the methods used to quantify experience have been varied – everything from in-lab questionnaires, school demographics, or neighborhoods demographics to quantify “typical” experience or exposure. While none of these methods are incorrect, they conflate close personal relationships with more distal properties of the social environment, which makes it difficult to tease apart which kinds of experiences contribute to children’s social cognitive development.

To better understand the nature and breadth of early social relationships, we developed a network questionnaire to extract infants’ and children’s early social networks, which will be referred to as *The Child Social Network Questionnaire (CSNQ)* for the rest of this paper. As described in more detail below, a child’s social network will refer to the people they interact with on a regular basis. The *CSNQ* will extract the following information for each child: (1) Network Size, or the number of people a child interacts with on a regular basis, (2) The diversity of social partners present in the network, measured with Entropy and EI Index (see Methods), and (3) Network Structure, or how the social relationships are patterned and connected in the social network (measured with Components, see Methods). Social networks provide a novel, innovative tool to operationalize early social experience for infants and young children. These properties can then be used to explore how experience relates to social cognitive development.

### The present study

The goals for this paper are twofold. First, we describe the *CSNQ* and the kinds of metrics that can be calculated for each child. We developed the *CSNQ* to collect social network data from children in infancy through early childhood. We collected data from the parents of children living in the US, predominantly in and around a large city. The *CSNQ* certainly is not exhaustive of all the network metrics that could be calculated for children; however, this paper focuses on the network metrics that map onto dimensions of early social experience that developmental psychologists typically care about.

Second, we provide a test case about how this questionnaire, and network methodology more broadly, can be used in developmental samples. In addition to providing descriptive information about children’s network variables and how they relate to each other, we will also ask the following questions: How do social networks vary with age? How is diversity assessed in the social network? How does network diversity vary with age and neighborhood demographics? The analysis presented below sheds light on the ways in which children’s social networks may vary across early development as well as how to contend with diversity in early social environments. We recruited 280 infants and children and provide a set of descriptive analyses, and we have made the dataset and analytic tools available on The Open Science Framework.^1^

## Materials and methods

### Participants

The participants were recruited in two places. The first group of participants (*n* = 209; *M*_age_ = 24.9 months; range: 6.4–59.1 months) were tested in a developmental laboratory in Chicago, IL; these were families from the city of Chicago and the surrounding suburbs who volunteered to be in a database for those interested in participating in early childhood research. The second group of participants was recruited at a paid to enter science museum in Chicago, IL (*n* = 108, *M*_age_ = 48.1 months; range: 36–59.4 months). A total of 37 subjects were excluded from the final data analysis due to experimenter error in conducting the interview (*n* = 30) or parents not being able to provide complete data during the parent interview (*n* = 7) for a final sample of 280 children (*M*_age_ = 33.3 months, range: 6.4–59.4 months). The museum is a tourist destination, so while 75% of our participants were from Chicago, IL and its surrounding suburbs, 25% were from other areas in the United States. Parents reported their children were 56.0% White or European-American, 15.2% Black or African-American, 7.1% Asian or Asian-American, 9.9% Hispanic or Latino/a-American, 19.9% mixed or biracial, and 3.2% as Other. For the laboratory-tested subjects, we recorded maternal education. 74.3% of those children had college-educated mothers.

### The child social network questionnaire

The *CSNQ* is administered in two parts: (1) a parent interview to collect information about children’s typical week of activities and (2) a form to collect demographic information for each person the child sees on a regular basis; this form is used to calculate the network measures described below. In network terminology, the parent interview is the “name generator” – this is the method used to elicit each of the people that should be included in the social network. The people in the social network are called “nodes” or “alters” (Robins, 2015). The demographic information is the “name interpreter” and this is the method used to collect the basic demographic information or other attributes of the alters (Perry et al., 2018).

#### Parent interview

Parents were asked to consider their child’s “typical week” of activities. The interview was explained as follows: “First, we will do an interview where I will ask you to describe [CHILD’s] typical week. We want to understand the different people [CHILD] sees in a typical week and what kinds of activities he/she does with those people. I am going to ask you about times [CHILD] wakes up, goes to sleep, and takes a nap so we can get a rough measure of the amount of time they spend with different people. After the interview, I will create a form for each of the people you mentioned to collect basic demographic information and also questions about how close you think your child is to that person. Starting with Monday, what time does your child wake up and what happens after that?” After parents described their child’s schedule, the experimenter asked, “Is there anyone else that you think is worth mentioning that your child sees on a regular basis?” Parents’ description of their child’s typical schedule served as a memory prompt and allowed the experimenter to make sure all the individuals a child regularly interacted with were accounted for (see Appendix A for details about the parent interview). This method of recall has been used to maximize the chances that respondents will fully report social contacts, and not omit the weaker ties in the social network (Small, 2017). After the parent interview, parents completed a demographic survey for each of the people in their child’s social network. Parents completed the demographic form in-person (*n* = 249) or in a follow-up, online form (*n* = 31).

#### Demographic form

There were two different versions of the demographic form for laboratory testing and public museum testing. Laboratory testing allowed for longer questionnaires to be administered to families. To accommodate the need for briefer sessions in the museum setting, the demographic form was shortened so the entire session only took 5–10 min to complete. For both laboratory and museum testing, the form asked for the following basic demographic information for each person: gender, age, race, and languages the person speaks. For laboratory testing only, the form collected information about the intensity of the individual’s relationship with the child (see Appendix B). For museum testing, we added questions about the different contexts or settings each person interacts with the child. This allowed us to infer relationships among the alters and compute the density, or how interconnected the network is, for each child (see Appendix C).

### Network variables

#### Network size

Network Size was defined as the total number of unique individuals and groups a child saw on a weekly basis. A parent had to report that the child knew the person as an individual for that person to be their own node. For example, if the parent reported that the child was in daycare or preschool, the experimenter would ask, “Are there any kids in the class that stand out as friends?” In addition to the individual named friend nodes, there would also be a node for “daycare/preschool class,” which is a node that includes multiple people. This distinction was made in order to capture the network of people that the child “knows” as individuals and about whom parents were likely to be able to report demographic data. For adults, the social network of an individual is a hierarchy that can be conceptualized as concentric circles (e.g., Hill and Dunbar, 2003); this method allowed us to capture the inner most circle for children. In the network science literature, the research question determines the boundaries of the network (Borgatti and Halgin, 2011). Social networks are most useful for developmental psychologists if they capture children’s recurring social contact; therefore, the network space we were interested in is who the child knows and has regular contact with.

#### High and low intense relationships

For the laboratory-based subjects (*n* = 161), there were three measures to assess the intensity of each relationship: the number of activities the person does with the child, how emotionally close parents reported their child feels toward the person, and the proportion of waking hours the person spends with the child (see Appendix B). A z-score was calculated across all 1122 relationships for each of the three measures and an average z-score was computed for each relationship. A median split of the average z-score then classified each relationship as either “low” or “high” intensity (see Supplementary Figure 4 for the distribution of z-scores for all the social relationships).

#### Proportion of kin and adult relationships

Each relationship was also classified as being kin or not kin. Kin is any relationship in the immediate and extended family (including grandparents, aunts, uncles, cousins, etc.). The proportion of kin relationships was calculated for each child’s social network: number of kin relationships/total Network Size. Each relationship was also classified as either being an “adult” or “child” relationship (child was anyone under the age of 13). The proportion of adult relationships was calculated as follows: number of adult relationships/total Network Size.

#### Network structure

##### Density

**Figure 1:**
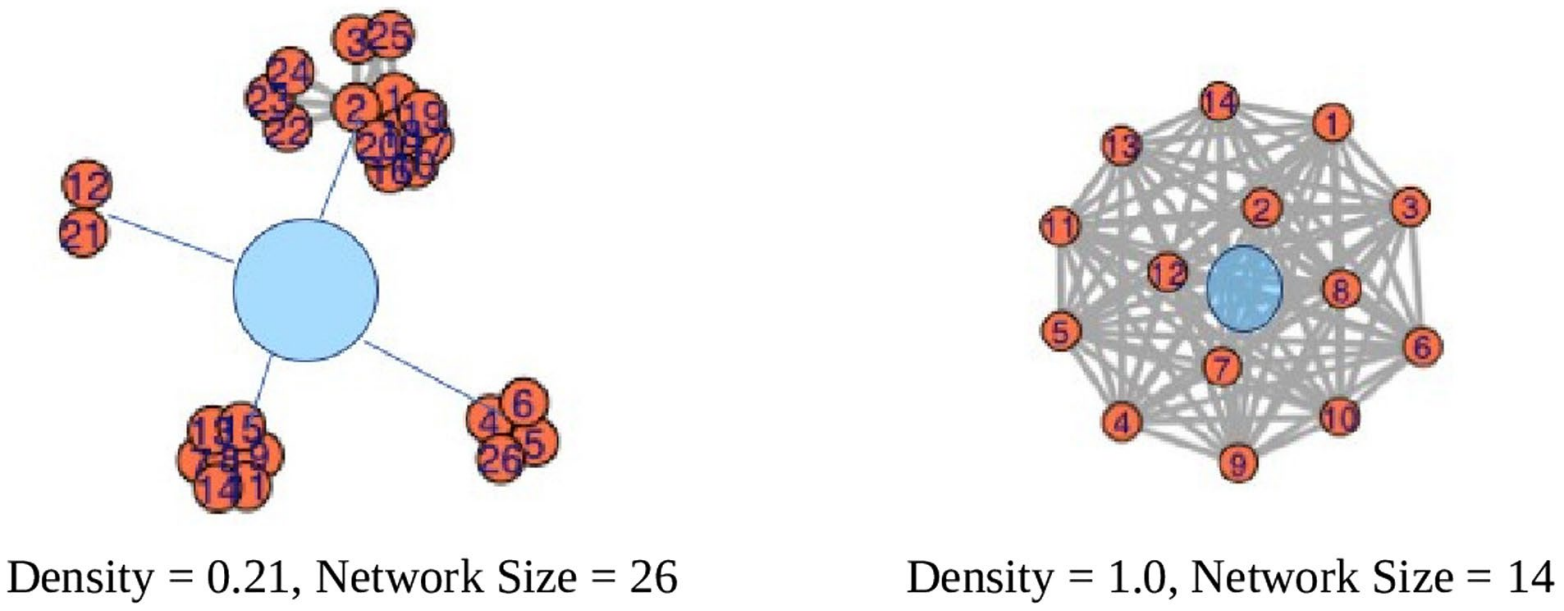
Example network graphs. The blue circles represent a child or “ego.” The lower the value, the less connected the social network.

The most basic structural measure of a social network is density. Density is a measure of the degree of connectedness of alters (i.e., who interacts with whom) in the network and was calculated as follows where *T* is the number of ties: Density = 2 *T*/*N*(*N* – 1) (Perry et al., 2018). A network where all the individuals know each other would have a density score of 1. A value <1 means that not all the alters know each other – the lower the number, the less connected the network. For the museum sample only (*n* = 101; *M*_age_ = 48.1 months; range: 36–59.4 months), we could calculate density because the *CSNQ* included a question asking about the different contexts that the individuals interacts in with the child. Example network graphs are presented below to understand what the density values represent visually (Figure 1).

##### Number of components

Another way to describe the structure of the network is the number of components. A component emerges in the network when all the alters are connected to each other in some way (Perry et al., 2018). Components are used in network science to assess how fragmented or spread out the network is in space. In an egocentric network, a component emerges when all the alters are connected even when the child is removed from the network. For example, imagine a child in a family who has a Mother, Father, and Sister. If you remove the child, the Mother, Father, and Sister all still interact with each other, which makes those relationships a single component. In Figure 1, the child on the left has four components and the child on the right has one component. In the adult literature, this is typically assessed by asking a person to report on all the pairwise relationships of who knows whom (Perry et al., 2018). Adding those questions to the *CSNQ* would have made the survey considerably longer and therefore more time consuming to administer in the laboratory along with child assessments. To assess the components in a child’s network, we asked about the different activities the child did throughout the week. The different activities were the components – for the activities, all the people at that activity would know each other. Every child has at least one component. Children with just one component only interacted with family members. Children with more than one component had a family and some other activity such as daycare, school, library story time, gym daycare center, ninja class, neighborhood potlucks, Sunday School, Chinese class, art class, or playgroup, just to name a few.

##### Component ratio

Finally, a social network can be described by how fragmented the network is in space. In Figure 1, the network on the left is more fragmented and spread out than the network on the right. The measure to describe how fragmented a network is called the Component Ratio. Larger networks tend to have more components, so to account for network size the Component Ratio is calculated as follows: (Components – 1)/(Network Size – 1) (Perry et al., 2018). Larger values of the component ratio indicate that the network is more fragmented. In Figure 1, the network on the left (Component Ratio = (4 − 1)/(26 − 1) = 0.12) is more fragmented than the network on the right (Component Ratio = (1 − 1)/(14 − 1) = 0).

#### Network diversity measures

In network science, there are two conceptually distinct ways to describe network diversity. The first measure describes the representation of different social categories present in the network, which is called entropy (Perry et al., 2018). The second measures indicates how diverse the network is relative to the child, which is called the EI Index (Krackhardt and Stern, 1988). Both entropy and the EI Index were calculated using the egor package in R (Krenz et al., 2020) and they were used to describe the diversity of two relevant social groups for American children – race and language.

##### Entropy

For network science, entropy indicates the relative presence of different social categories among the alters in a network and is calculated as follows for a given probability vector of *P*(*X*): *H*(*X*) = −∑ *P*(*X*) * log2(*P*(*X*)) (Drost, 2018). The probability vector is the proportional representation of different social categories. For example, if half the alters in the network were Black and half the alters were White, then the probability vector would be *X* = (0.5, 0.5). If half the alters in the network were White, 25% were Black, and 25% were Asian, then the probability vector would be *X* = (0.5, 0.25, 0.25). An entropy score of 0 indicates that there is no diversity of categories; all the alters share the same attribute (e.g., all the alters are the same race). A higher entropy scores indicates a greater representation of different categories and the more categories present (i.e., the more racial groups present in the network) the higher the entropy score. For the example networks given above, the network where half the alters were White and half were Black, would get an entropy score of 1. For the network with White, Black, and Asian alters, the entropy would be 1.5. The more groups or categories represented, the higher the entropy.

##### Network racial entropy

To calculate racial entropy, each alter was classified by a discrete racial category. The racial categories that were used to calculate entropy were the following: African or Black-American, Asian or Asian-American, European or White-American, Hispanic or Latino-American, Native American, Mixed/Biracial, or Other. For the Mixed/Biracial category, parents could indicate that the alter was biracial by selecting “Mixed/Biracial” or by selecting more than one race. For some alters, we have detailed information (for example, if the alter was a Black/White biracial or Asian/White biracial), but for some alters we only know that they are biracial. As such, all biracial alters were categorized as “Mixed/Biracial.” This is imperfect as biracial individuals are not a monolith; however, this method of categorization allowed us to retain all the racial information about the alters. See Supplementary Figure 5 for visual representations of different racial entropy scores.

##### Network language entropy

Similar to the racial entropy, each alter was categorized to fit into a discrete language category to calculate language entropy. This is a primarily English-speaking sample; all the children were recruited to participate in studies in English and required that English be spoken at home at least 50% of the time. The most dominant language category was monolingual English speakers (66.3% of all alters), followed by English bilingual speakers (22.5% of all alters), preverbal infants (1.7% of all alters), and non-English monolingual speakers (1.0% of all alters). Language data was missing for 8.5% of the alters and they were excluded from analysis.

##### EI index

The EI Index is a measure of homophily the child shares with the network and is calculated as follows: (Number of Different Alters − Number of Same Alters)/Network Size (Krackhardt and Stern, 1988). The EI Index ranges from −1 to 1; a score of −1 indicates the entire network is the same as the child on some attribute and a score of 1 indicates that the entire network is different from the child on some attribute. For example, if a White child had a network where all the alters were White, they would have a score of −1; see Supplementary Figure 6 for visual representations of the EI Index.

##### Racial EI index

To calculate the racial EI Index, each alter was classified as either same-race or different-race compared to the child. For monoracial children, this was simple – any alter that was not the same race as the child was coded as different-race (i.e., for a White child, any alter that was not also White was coded as different-race). For biracial children (19% of our sample; *n* = 51), the alter was classified as same-race if they were of either races of the child. For example, for a Black/White biracial child, any alter that was White or Black would be coded as same-race. All other alters would be coded as different-race. For biracial children, parents either provided detailed information for their child or we deduced the races of the child by examining the races the parents reported for themselves.

##### Linguistic EI index

For the Linguistic EI Index, each alter was coded as same-speaker or different-speaker. For monolingual English children, this meant anyone who spoke a language other than English was coded as different-speaker. For bilingual and multi-lingual children, an alter was coded as different-speaker if that person spoke a language the child did not speak. For example, imagine an English/Spanish bilingual child with a network where 2 people spoke English, 1 spoke English and Spanish, and one spoke English and Dutch. The only alter that is a different-speaker is the English/Dutch bilingual because the child does not speak Dutch and would therefore have a Linguistic EI Index of −0.5 ([1–3]/4).

### Neighborhood demographics

In addition to completing the *CSNQ*, parents also provided their zip code. Using data from the US Census (US Census Bureau, 2018), we extracted Neighborhood Racial Entropy and Neighborhood Linguistic Entropy for each child (Hwang, 2018). 65% of the sample lived in an urban setting with a median income of $68,770 (range: $28,965–$196,964).

## Results

The results presented below will accomplish the following aims. First, we present the descriptive information about the network variables and how they related to each other, which will highlight the ways in which children’s social worlds vary by the size of the network. Next, we will answer the following questions: How do social networks vary with age? How is diversity assessed in the social network? How does network diversity vary with age and neighborhood demographics? Social network data tends to be skewed and colinear, given the nature of social phenomenon (Perry et al., 2018); therefore, we used non-parametric analyses for network variables that were not normally distributed.

### Social network variables

Table 1 shows the mean, standard deviation, and range for the following network variables of interest: Network Size, Raw Number of Low and High Intense Relationships, Proportion of High Intense Relationships, Proportion of Kin Relationships, Proportion of Adult Relationships, Density, Number of Components, Component Ratio, Racial Entropy, Racial EI Index, Language Entropy, Language EI Index. For visual examples of the network structure values, refer to Figure 1.

**Table 1:** Table of the social network variables.

	Mean (SD)	Range
Network size	11.0 (5.0)	3–27
Raw number of low intense relationships	3.9 (3.2)	0–15
Raw number of high intense relationships	3.8 (2.0)	1–12
Proportion of high intense relationships	0.54 (0.23)	0.13–1.0
Proportion of kin relationships	0.52 (0.24)	0.05–1.0
Proportion of adult relationships	0.65 (0.18)	0.05–1.0
**Network structure**		
Density	0.56 (0.21)	0.21–1.0
Number of components	2.5 (1.2)	1–7
Component ratio	0.15 (0.12)	0–0.67
**Diversity measures**		
Racial entropy	0.91 (0.62)	0–2.4
Racial EI index	−0.51 (0.45)	−1–0.8
Language entropy	0.69 (0.44)	0–1.8
Language EI index	−0.76 (0.29)	−1–0.2

#### How do the network variables correlate with network size?

The most fundamental part of a social network is social network size (Perry et al., 2018). As network size increases, other aspects of the network tend to covary as well. Network structure measures are inherently linked to network size – as network size increases the number of components typically increases while the density of the network decreases (Perry et al., 2018). Table 2 presents FDR-corrected correlations between Network Size and the other network properties. Consistent with the adult social network literature, network size covaried with network structure – as the network size increased, the number of components increased (*rho* = 0.63, *p* < 0.001) and the density of the network decreased (*rho* = −0.52, *p* < 0.001). As network size grew, children had more contexts that they interacted in and the connectedness of the network decreased. The content of the network also covaried with size. As network size increased, the proportion of high intense relationships (*rho* = −0.41, *p* < 0.001), the proportion of kin relationships (*rho* = −0.56, *p* < 0.001), and the proportion of adult relationships (*rho* = −0.38, *p* < 0.001) all decreased. As network size grew, children interacted with more low intense relationships, more children, and more people outside of their family. Finally, the diversity measures also covaried with network size. As network size increased, the racial entropy (*rho* = 0.35, *p* < 0.001), racial EI Index (0.24, *p* < 0.001), and linguistic EI Index (0.24, *p* < 0.001) increased as well. As network size increased, so did the various measures of diversity.

**Table 2:** Table of correlations between network size, age, and other network variables.

	Network size	Child age
	Spearman rho	Spearman rho
Network size	–	**0.61** ^***^
Child age	**0.61** ^***^	–
Prop high intense relationships	**−0.41** ^***^	−0.03
Proportion of kin relationships	**−0.56** ^***^	**−0.41** ^***^
Proportion of adult relationships	**−0.38** ^***^	**−0.42** ^***^
Density	**−0.52** ^***^	−0.19
Number of components	**0.63** ^***^	**0.63** ^***^
Component ratio	−0.01	0.04
Racial entropy	**0.35** ^***^	**0.25** ^**^
Racial EI index	**0.24** ^***^	**0.16** ^*^
Language entropy	−0.01	−0.07
Language EI index	**0.24** ^***^	0.09

*** *p* < 0.001;

** *p* < 0.01;

* *p* < 0.05.

This correlational analysis demonstrates that social environments and social phenomenon are complex and embedded. While several of these dimensions of early social experience are conceptually distinct, this analysis shows they can also be empirically related. When using social network analysis and theory as a framework to understand how social experience relates to development, it is necessary to understand which aspects of experience covary to be precise about which aspects of experience relate to social cognitive outcomes. If developmentalists only focus on one dimension of experience without measuring other aspects of experience, it is impossible to know what precisely contributes to development.

### How do social networks vary with age?

Descriptive social network data collected over a wide developmental age range can answer the question: How do early social environments vary with age? The analysis presented below demonstrates how network properties, which describe early social environments, vary across developmental time. The analysis presented below used FDR correction for multiple comparisons and we present the correlations in Table 2 (for scatterplots with all the network variables and child age, see Supplementary material).

#### Network size and age

**Figure 2:**
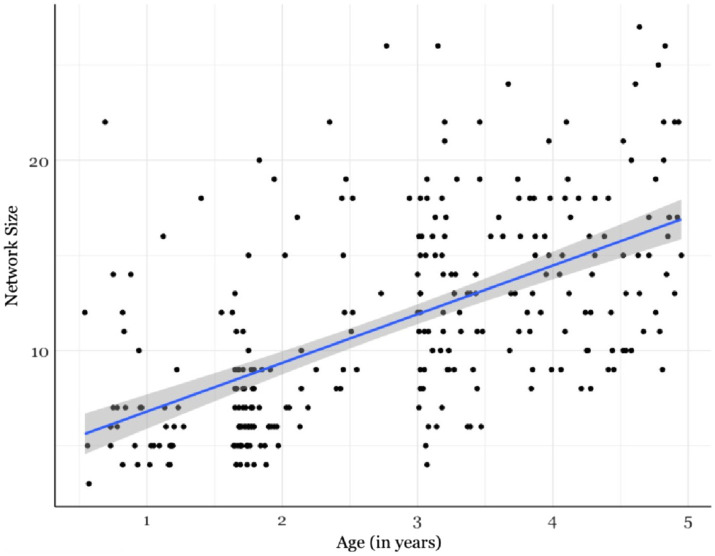
There was a significant, positive correlation between Network Size and child age; as children got older their Network Size increased (*rho* = 0.61, *p* < 0.001). Shaded area indicates the 95% confidence interval.

Network Size was correlated with children’s age to explore how the number of people a child interacted with on a regular basis varied across the first few years of life. Network Size was square root +0.5 transformed because Network Size is a small count variable (Kirk, 2013). The results showed a significant, positive correlation between Network Size and age; as children got older their Network Size increased (*rho* = 0.61, *p* < 0.001; Figure 2). At a time when children are experiencing rapid changes to their social cognitive development, they are also experiencing drastic changes to their early social environments. The number of close, reoccurring social relationships children had increased over the first few years of life.

This growth in network size cannot be entirely be explained by children entering school. A linear regression was conducted to test the effects of child age, out-of-home childcare, and their interaction on Network Size; the regression was significant (*R*^2^ = 0.39, *F*(3, 275) = 61.3, *p* < 0.001). There was a main effect of age (*β* = 0.02, *p* < 0.001), no main effect of childcare experience (*β* = 0.10, *p* = 0.62), and no significant interaction (*β* = 0.006, *p* = 0.28; see Supplementary material). Although it is true that children in out-of-home childcare had larger social networks than children without out-of-home childcare (*M*_OutofHomeChildcare_ = 13 people (5), *M*_NoOutofHomeChildcare_ = 9 people (5); *W* = 5,031, *p* < 0.001), when controlling for the effect of out-of-home childcare on Network Size, child age was a significant predictor. Post-hoc, Bonferroni corrected correlations were performed to explore if the age trend is present for both children with and without out-of-home childcare and there was a significant, positive correlation with age for both groups of children (Out-of-home childcare: *r* = 0.61, *p* < 0.001; No Out-of-home childcare: *r* = 0.41, *p* < 0.001). Regardless of childcare experience, as children got older their networks grew.

In addition to the tremendous growth in size during the first few years of development, there was also substantial variation at any given time point. This variability was present in infancy and continued throughout early childhood. Taken together, this raises two interesting possibilities. First, at a time when children see a rapid expansion in the number of social relationships they interact with on a regular basis, they are also experiencing rapid changes to their social cognition. Their social cognitive skills start to emerge and mature during the first few years of life, which raises questions about how the growth in network size relates to the emergence and development of these skills. Second, while there is steady growth in network size, there is also substantial variability at any given age during this developmental window. This variability opens up questions about how variation in network size relates to variation in social cognitive skill development – these are questions that can be asked in infancy and throughout early childhood. As networks grow, this creates the possibility for changes in other aspects of network structure. The next analysis evaluated age-related variation in network composition and structure.

#### Network composition and age

The next set of analyses explored how network composition varied with child age. Network composition refers to the make-up of the social network – the high and low intensity relationships, the kin relationships, and the age of relationships. Before exploring how network composition varied with child age, we explored the nature of kin relationships and whether these were also the high intensity relationships. Across all participants, there were a total of 1,122 social relationships that could be classified as either kin or not kin (*n*_kin_ = 683, *n*_notkin_ = 439) and high or low intensity (*n*_highintensity_ = 561, *n*_lowintensity_ = 561). On average, approximately half of children’s relationships were high intense and half the relationships were kin (Table 1); however, not all kin relationships were necessarily high intense. 31% of the kin relationships were low intensity relationships (*n* = 212) and 20% of the not kin relationships were high intensity relationships (*n* = 90). Children had some non-kin relationships that were high intense relationships, such as daycare teachers, and they had kin relationships that were low intense, such as extended family members. We also looked to see if the Proportion of Kin relationships was correlated with the Proportion of High Intense relationships. Networks with a larger proportion of kin relationships were also networks that had a larger proportion of high intense relationships (*rho* = 0.42, *p* < 0.001).

We next explored how the proportion of high and low intensity relationships, kin relationships, and adult relationships varied with child age. The FDR-corrected correlations are reported below and displayed in Table 2. The high intensity relationships (*rho* = −0.03, *p* = 0.72) and the proportion of low intensity relationships (*rho* = 0.03, *p* = 0.72) were not correlated with age. Both proportion of kin relationships (*rho* = −0.41, *p* < 0.001) and proportion of adult relationships (*rho* = −0.42, *p* < 0.001) were negatively correlated with age.

While there was a relation between child age and proportion of kin relationships and adult relationships, there was no evidence that the proportion of high intense relationships was related to child age. This is surprising because the proportion of kin relationships was positively correlated with the proportion of high intense relationships. Taken together, this suggests that kin relationships were not the only source of high intense relationships in early childhood. As children got older, they started to interact with more people outside their family, but these people could still be high intense relationships, such as a teacher or a close friend. It was also true that as children got older, they started to interact with more children and similarly-aged peers. These analyses suggest that as children get older, the composition of their social world undergoes significant changes, which opens up questions about how changes in the nature of social interaction impact cognitive development.

#### Network structure and age

We next explored how network structure varied with child age. This analysis focused on density, components, and component ratio. Density is the most basic structural aspect of a network and describes the extent that people in the network are connected to each other. Components describe the different contexts that children interact in and Component Ratio describes how fragmented in space the network is.

##### Density and age

There was no significant correlation between density and child age; there was no evidence to suggest that as children got older their networks become less connected (*rho* = −0.19, *p* = 0.12; Table 2). Importantly, density could only be calculated for data collected at the museum (*n* = 101), which reflected a smaller age range than the rest of our sample. It is possible the null result is due to the constricted age range.

##### Components, component ratio, and age

To explore how network structure related to age, we next looked to see how the number of components and the Component Ratio correlated with age. The number of components was positively correlated with age – as children got older, the number of components in their network increased (*rho* = 0.63, *p* < 0.001; Table 2). Interestingly, the Component Ratio was not correlated with age; there was no evidence that the fragmentation of children’s networks varied with age (*rho* = 0.04, *p* = 0.59; Table 2). It is possible the Component Ratio stayed relatively flat throughout the first few years of development because while it was true that the number of components increased over developmental time, so did network size. The Component Ratio was calculated with Network Size in the denominator (Number of Components − 1/Network Size − 1), which explains why the relative fragmentation stayed consistent throughout the first few years of life when both the components and network size were rapidly growing. See Supplementary Figures 13, 14 for the scatterplots for Network Structure and child age.

#### Summary of age findings

Our results showed compelling evidence that as children got older, their network size, or the number of people they interacted on a weekly basis, increased. At a time when children’s social cognitive skills are rapidly emerging and developing, they are also experiencing drastic changes to their social world. In addition to an increase in the number of people children saw on a weekly basis, there was a decrease in the proportion of kin and proportion of adult relationships. As children got older, they interacted with more people outside of their immediate family and started to interact with more children and peers. Not only did the number of people who children interacted with changed as they got older, the kinds of people they interacted with changed as well. This raises interesting questions about the role that non-kin and other similar-aged peers play in children’s development. Prior developmental work has emphasized the role of parent–child interactions for early development; however, the results presented here showed that children have relationships with a broader network. It is fruitful to consider the value of these other relationships for children’s cognitive development.

### Diversity in social networks

The final set of analyses explored the ways that diversity can be measured in social networks in early childhood, how network diversity varies across the first few years of life, and how network diversity relates to broader neighborhood demographics. Although network measures can be used to describe the diversity of any attribute that can be measured about a person, this paper focused on two social categories relevant to American children – race and language. Both racial and linguistic diversity were assessed using entropy, which describes the representation of different social groups, and EI Index, which describes how diverse the network is relative to the child.

Before exploring how network diversity varied by age or by neighborhood demographics, the entropy and EI Index measures were correlated with each other using Spearman correlations. Both measures of racial diversity were correlated with each other (*rho* = 0.72, *p* < 0.001) as were both measures of linguistic diversity (*rho* = 0.50, *p* < 0.001). Although these are conceptually distinct ways to operationalize diversity in a social network, in early childhood, these measures are highly correlated with each other.

#### Network diversity and age

There was a significant positive correlation with age for Network Racial Entropy (*rho* = 0.25, *p* < 0.001) and for Network Racial EI Index (*rho* = 0.16, *p* = 0.01; Table 2). As children got older, the representation of different racial groups in their network increased, as did the amount of racial outgroup members. There was no significant correlation between Network Language Entropy and child age (*rho* = −0.07, *p* = 0.29) nor between Network Linguistic EI Index and child age (*rho* = 0.09, *p* = 0.28; Table 2). Unlike Network Racial Diversity, there was no evidence that Network Language Diversity changed as children got older.

In addition to increased network size with child age, there was also evidence that network racial diversity increased with age. The representation of different racial groups in their network and how diverse the network was relative to the child’s own race, increased with child age. Interestingly, there was no evidence that network language diversity was related to age; neither language entropy nor the linguistic EI index were correlated with child age. These set of findings have implications for how developmental psychologists should consider the effects of diversity on children’s emerging social cognitive abilities. Data from US children suggest that as children get older, they are exposed to more racial groups, but they are not necessarily exposed to more different-language speakers.

#### How does network diversity interact with structural network properties?

The benefit of social network analysis is that it can be used to describe how network variables are related to each other. For instance, the network racial diversity can be described by using the two measures outlined above – racial entropy and racial EI Index. These measures perfectly describe the racial diversity of children’s reoccurring social contacts. Social network analysis can take this one step further to ask: How are different racial group members patterned in the social network? Is children’s contact with racial outgroup members interconnected or more dispersed in the social network?

Using the data collected at the museum (*n* = 101; *M*_age_ = 48.1 months; range: 36–59.4 months), we calculated not only the racial entropy of the network, but also the racial entropy of each of the components. Children ranged in the number of components they had – from 1 to 7 – and for each child, we could calculate the proportion of their components that had 0 entropy. A component that had 0 entropy meant that all the people in that component were the same race. A child with a proportion of 1 would mean all the components in their network had a racial entropy score of 0, which would indicate no network diversity. Although it would be theoretically possible for someone to have two 0 entropy components of different races and an overall network racial entropy >0 (i.e., a child has one component with all White members and one component with all Black members and therefore both components have 0 entropy), that did not occur in this dataset. Children that had a proportion of 1 were children in no diversity networks. A child with a proportion of 0 would mean that all the components had a racial entropy >0 – all the components had people of different races. The average proportion of 0 entropy components per child was 0.31 (SD = 0.30, range: 0–1).

Once the racial entropy of each component was calculated, it was then possible to identify different patterns of diversity that emerged. Table 3 highlights examples where subjects had identical overall Network Racial Entropy, but the racial diversity was patterned differently in the network. For example, Subject1 and Subject2 had the same overall Network Racial Entropy; however, Subject1 had a network where the racial diversity was not evenly distributed. Their family did not provide any racial diversity, but they had fairly high racial diversity at school. On the other hand, Subject2 had high levels of racial diversity across all their components; their social network was more racially integrated. Table 3 highlights that the Network Racial Entropy glosses over complexity present in children’s social relationships. Not only can the network be described by the composition of different social groups, but networks can also be used to explore how the pattern of those relationships might matter and impact social cognitive development. How racial outgroup exposure is patterned in their network is data to children about how the social world operates and likely informs their early intergroup cognition.

**Table 3:** Example social network information.

	Network size	Network racial entropy	# of components	Family component	School component	Other component 1	Other component 2
Subject ID							
Sub1	9	1.22	2	0.00	1.5	–	–
Sub2	18	1.22	4	1.25	1.22	1.52	1.56
Sub3	14	1.52	4	1.49	0.81	1.49	0.81
Sub4	10	1.52	2	0.00	1.41	–	–
Sub5	9	0.50	3	0.00	0.92	0.00	–
Sub6	9	0.50	2	0.00	0.65	–	–
Sub7	9	0.50	2	0.00	0.72	–	–

Children’s networks could further be characterized by how the racial diversity was distributed in the network – did children experience racial diversity in an integrated network, where there was non-zero entropy in each component, or did children experience racial diversity in a segregated way, where some components had no racial diversity and other components did? Each child’s network could be described as either integrated, segregated, or no-diversity networks. Integrated networks meant that the overall network racial entropy was greater than zero and each component in the network also had network racial entropy greater than zero. A segregated network was when the overall network racial entropy was greater than zero and the proportion of zero entropy components in the network was greater than or equal to 0.5, but <1 (see Supplementary material for visual examples). A no-diversity network meant that the overall network racial entropy was 0 – all the people in the child’s network were the same race and therefore each component also had 0 racial entropy. For this sample, 40% of the children had an integrated network, 54% had a segregated network, and 6% had no-diversity networks. This sample of children demonstrated that racial diversity can be patterned in several different ways. This raises the interesting question about whether *how* racial outgroup members are patterned in the social network matters for children’s emerging intergroup cognition.

#### How do network and neighborhood diversity relate to each other?

The final set of analyses explored how network and neighborhood diversity measures, specifically racial and linguistic diversity, related to each other. Prior developmental work has used neighborhood demographics to approximate experience (Weisman et al., 2015; Mandalaywala et al., 2019), but only one study has used neighborhood demographics to test how distal social experience affects cognition (Howard et al., 2014). It remains an open question whether neighborhood demographics have a differential impact on children’s cognition than the demographics of children’s reoccurring social contact. It is not well understood whether the neighborhood demographics provide different information to children than the demographics of their recurring social contact. Using children’s social network data and the US Census data, we can explore this possibility. Participants provided their zip code and their neighborhood racial and language entropy was calculated using the American Community Census Survey from 2018.

##### Network and neighborhood racial diversity

**Figure 3:**
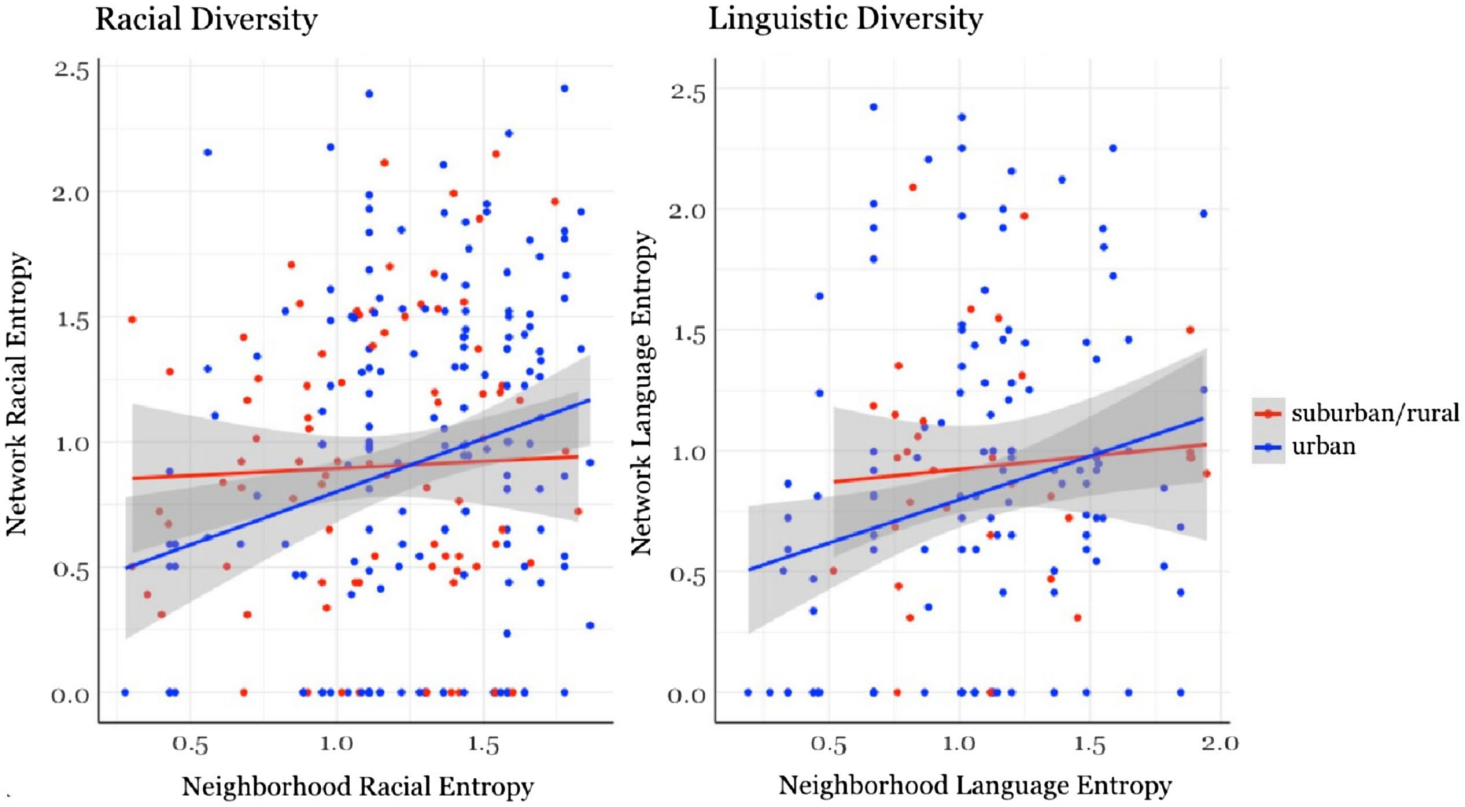
For urban subjects only, there was a positive correlation between Network and Neighborhood Racial Diversity (*rho* = 0.24, *p* = 0.002) and Network and Neighborhood Linguistic Diversity (*rho* = 0.25, *p* = 0.004). Shaded area indicates the 95% confidence interval.

The FDR-corrected correlation between Network Racial Diversity and Neighborhood Racial Diversity was positive and significant (*rho* = 0.17, *p* < 0.005). We further explored whether this varied by the geographic location – either urban or suburban and rural areas. Using the zip code, each participant was classified as either living in an urban area or suburban or rural area according to the CDC’s classification of counties (Ingram and Franco, 2014). Participants who lived in Cook county, but not in Chicago, IL, were classified as living in a suburban area. In our sample, 183 subjects lived in an urban area and 90 subjects lived in a suburban or rural area. Figure 3 shows Network and Neighborhood Racial Entropy by urban and suburban or rural areas. Spearman correlations revealed that for urban subjects only, there was a positive correlation between Network and Neighborhood Racial Diversity (*rho* = 0.24, *p* = 0.002), but there was no significant correlation for suburban or rural subjects (*rho* = 0.02, *p* = 0.89).

##### Network and neighborhood language diversity

Similar to the Racial Diversity findings, Network and Neighborhood Language Entropy were positively correlated with each other (*rho* = 0.22, *p* = 0.004). The geographic analysis showed the same pattern of results as the Racial Diversity findings. There was no evidence that Network and Neighborhood Language Entropy were related in suburban and rural areas (*rho* = 0.07, *p* = 0.71), but there was a positive correlation for urban areas (*rho* = 0.25, *p* = 0.004; Figure 3).

##### Summary of neighborhood findings

For both Racial and Language Diversity, network and neighborhood entropy were correlated with each other; however, this finding seemed to be driven by the subjects living in urban areas. For subjects living in suburban and rural areas, there was no evidence that their network and neighborhood Racial and Language Diversity were correlated. It is possible there was no evidence that network and neighborhood demographics map onto each other for suburban and rural areas because these areas are not as densely populated. Even if a suburban area has higher levels of Neighborhood Racial Entropy, that diversity could be spread out across greater areas of land than in urban areas. Most of the urban sample is from [Chicago, IL], which is a densely populated city and it is therefore reasonable that children’s reoccurring relationships would match the demographics of their community. Future work can explore why network and neighborhood demographics do or do not map onto each other; it is possible this effect could be explained by parents’ values about community involvement or the extent that their own social networks reflect the demographics of the communities that they live in.

Although these findings leave several open questions, this initial analysis suggests that networks and neighborhoods can supply different demographic information to young children, which raises important methodological and theoretical implications. First, this analysis shows that networks can be used to improve methodological practices in the field by better operationalizing children’s early social environments. Given that networks can be used to better describe early social environments and make a distinction between children’s close, reoccurring contact and their more distal social environment, we can then use this framing to generate questions about whether and how networks and neighborhoods have differential impacts on cognition.

## Discussion

This paper has demonstrated that a social network perspective can be used in developmental science to measure children’s early social environments. A social network perspective is not only helpful to describe early social environments, but once early social experience is conceptualized as network properties, a social network perspective can be used as a framework to generate hypotheses about how early social experience impacts cognition and development. For example, researchers can ask whether the number of individuals a child interacts with on a weekly basis impact their social cognitive skills. The data presented in this paper provide an initial look at how a social network perspective can be used in developmental science and raises several interesting implications for studying and understanding children’s early social environments.

Children’s social network data illustrate the varied aspects of social network structure that can be identified in infancy and early childhood. As the results showed, while several of these network variables and aspects of early social environments are conceptually distinct, they might also be empirically correlated with each other. To best understand how early social environments relate to children’s development, it is important to understand the aspects of early social environments that are related to each other. We presented analyses about how aspects of experience were correlated for this particular highly educated, urban sample in the US; however, it is possible, and in fact likely, that different patterns and trends would emerge for different samples of children, both cross-culturally and within the US. A social network perspective is useful for developmental science because different dimensions of networks can be used to describe social environments, but it is also useful because it provides a framework to understand the embedded and complex nature of early social environments.

Our results also highlight the ways in which children’s social networks vary across infancy and early childhood. Most notably, our cross-sectional analysis showed that as child age increased, so did network size. This was true across early development, during infancy, and continuing into the preschool years. Children’s networks also varied in other ways across the first few years of development. As child age increased, children interacted with more peers and less family, as well as interacted with more racial outgroup members. At a time when core aspects of social cognition are developing, children’s social networks undergo significant change. This raises the obvious question of whether and how social cognitive development may be affected by changing social environments, and raises the possibility that developments that were assumed to reflect maturation may instead, or in addition, be driven by experience.

Finally, our results demonstrate the ways in which networks can be used to assess and explore diversity in early childhood. Our results showed that while diversity can be evaluated in conceptually distinct ways, they are often related to each other in early childhood. This measure of precision in capturing and describing diversity will allow developmentalists to refine theories about how outgroup exposure affects social cognitive development. In addition to using social networks to precisely describe diversity, our results show the ways that diversity varies across age and geographic location. For racial diversity only, as child age increased, so did both measures of racial diversity. As children get older, they interact with more racial outgroup members. For subjects in urban settings only, both network and neighborhood measures of diversity were related to each other. This analysis demonstrated two important points. First, given that network and neighborhood diversity measures are not necessarily correlated with each other, it is problematic to use neighborhood demographics to approximate typical experience and pushes against assumptions that characteristics of a neighborhood would be reflected in children’s immediate social environments. Second, this analysis highlighted that networks and neighborhoods can provide different information to children; this raises the possibility that proximal and distal social environments have differential effects on cognition. Future work can probe this possibility to refine theories about how experience shapes cognition.

It is important to reiterate that our sample is highly educated (over 70% of mother’s have a bachelor’s degree or higher), approximately half White, mostly reside in or around a large, urban city, and data was collected before the COVID-19 pandemic; it is necessary to test widely to explore whether these findings generalize across different samples. As stated above it is possible, and likely, that these patterns vary across different samples, even within the US. In particular with socioeconomic (SES) diversity, there are theories in the network literature that suggest adults from high- versus low-SES backgrounds have different functions for the high and low intensity relationships in their networks (Granovetter, 1983; McPherson et al., 2006). Indeed, there is a general trend in the US that adults with higher levels of education have less kin relationships in their social networks (McPherson et al., 2006). Future work will need to explore whether these trajectories of early social experience are the same for children from low-SES backgrounds.

Prior developmental work that has implemented network methodology has used methods of validation to quantify the networks. For example, in a study asking children about who their friends were in a classroom, researchers also surveyed the teacher to see if the reported friendships by the children were accurate (Neal et al., 2016). Our method relied on parent report, which is a commonly used method for the reported age range because infants and young children cannot reliably provide the information themselves. Parent report was chosen to elicit young children’s networks because parents are the most reliable informants about who their child sees on a regular basis. Especially in infancy, someone is always watching the infant and American parents always know who that person is. It is important to note that parent report is not without limitation, especially as children get older and gain more autonomy in who they spend time with. For example, there is a reason parent report is not used to extract adolescent social network data. While children could have been observed more naturalistically, this method of recall is commonly used in the adult social network literature, so it made sense to adapt that protocol for this particular developmental sample. Further, by asking a more objective question about who the child has contact with rather than something more subjective like “name your children’s friends,” we can eliminate some of the bias that contributes to mismatches between who people report their friends are and who their actual friends are.

### A network perspective in developmental science

Social network research can be broken down to research questions that study network variables as predictors, outcomes, or both – this produces three distinct theoretical approaches to the study of social networks (Borgatti and Halgin, 2011; Perry et al., 2018). Network variables as predictors has been the focus of this paper; however, other theoretical network approaches can be used to generate cutting-edge questions for the field of developmental science.

Studies that explore network variables as outcomes ask questions about how a non-network variable leads to the formation of the social network. For example, do parents’ values about diversity relate to the racial make-up of children’s network? Do parents’ values about diversity have an indirect effect on children’s racial bias as a result of the child’s social network? How do parents’ beliefs about family relate to the formation of children’s networks? Rather than focusing on how network properties might relate to social cognitive development, questions under this theoretical approach can be used to ask how non-network aspects of early social experience (such as attending school) might relate to the formation of certain social networks.

Studies that explore network variables as both outcomes and predictors ask questions about how a particular network phenomenon relates to another network phenomenon. Research questions that employ longitudinal social network design would fall under this theoretical approach. Although there is obvious value in using social networks in a cross-sectional design, social networks are an exceptional tool to ask how *changes* in network properties relate to *changes* in cognition or behavior. For example, the cross-sectional analysis showed that child age was correlated with network size. Is it the case that as children get older, their network size increases? Does an increase in network size relate to changes in social cognitive abilities? The cross-sectional data suggests that children do experience growth in their networks during the first few years of life, which would allow developmentalists to test whether changes in network size relate to changes in social cognitive capacities. Prior work with adults and non-human primates suggests that this might be true – those with larger social networks have superior social cognitive skills (Stiller and Dunbar, 2007) as well as changes and increases to brain size and function (Bickart et al., 2011; Sallet et al., 2011). These questions can be explored further using the *CSNQ* in a longitudinal design.

### Recommendations for developmentalists who want to use social network analysis

Developmentalists have nuanced theories about the kind of experiences that might be important for shaping children’s early social cognition. However, until now, there have not been the proper tools to capture the whole picture of infants’ and young children’s early social interactions. Social networks are a powerful tool that can begin to address prior debates in the field as well as inform cutting age theories about social cognitive development.

To most effectively use network analysis and methods, it is necessary to be intentional about applying social network analysis to developmental questions. Social network analysis is a powerful tool and it is not a method that can be applied thoughtlessly. Social network theory will generate several hypotheses about how early social environments affect social cognition; however, it is crucial to specify which kinds of experience might matter for development. Social network theory can be used as the framework to consider how certain aspects of experience might be correlated with each other; this framework and school of thought can then lead to thoughtful experimental design to tease apart which kinds of experience might matter for development. Relatedly, it is important to not overinterpret data and to be clear about what claims can and cannot be made from social network research. Early social environments are complex and embedded, and social network research is largely correlational; it is necessary to be clear and honest about what conclusions can be drawn from the data.

The *CSNQ* used social network methods to capture and describe the people children interact with on a regular basis, but children do not have one social network. Several different kinds of networks can be extracted for an individual; the network space is determined by the research question (Borgatti and Halgin, 2011). For each of the dimensions of social networks, a researcher can make different decisions about what kind of network will be examined. For example, instead of asking about who the child interacts with during a typical week, a researcher could ask about every individual the child saw in the last month, which would cast a wider network space of people to be included. The questions that are asked about each of the alters can also vary. The *CSNQ* focused on racial and linguistic network diversity, but a researcher could ask an endless amount of questions about each of the alters: religious affiliation, political identity, education level, food preferences, or even shared beliefs. Finally, the kind of relationships that are examined could vary. The *CSNQ* focused on who the child “knew” and saw regularly, but the relationships that are examined could be affective ones (who the child “likes” or “dislikes”) or even event-based interactions, such as how many times a child plays with another child (Borgatti and Halgin, 2011). We made decisions across these three dimensions to make the *CSNQ* most useful to developmentalists; however, network methods can be adjusted to extract different kinds of networks based on the desired research question.

In summary, the *CSNQ* is an excellent tool to capture and describe children’s early social relationships. When early social environments are conceptualized in a unified framework, social network theory can be used to generate questions and hypotheses about how experience impacts social cognitive development. A network perspective will expand and explore these kinds of questions, which will allow researchers to produce hypotheses about the mechanisms underlying early social experience. The data presented here offers initial insight into the potential usefulness of this framework for developmental research, providing both a tool and a conceptual framework to better explore the nature of early social environments and their potential relations to social cognitive development.

## Data availability statement

The original contributions presented in the study are publicly available. This data can be found at: https://osf.io/3hc7n/?view_only=49848537a6c543d7807020537d5da0b0.

## Ethics statement

The studies involving human participants were reviewed and approved by University of Chicago IRB. Written informed consent to participate in this study was provided by the participants’ legal guardian/next of kin.

## Author contributions

All authors listed have made a substantial, direct, and intellectual contribution to the work and approved it for publication.

## Funding

This research was supported by an NIH grant (P01 HD064653) awarded to Dr. Amanda Woodward.

## Conflict of interest

The authors declare that the research was conducted in the absence of any commercial or financial relationships that could be construed as a potential conflict of interest.

## Publisher’s note

All claims expressed in this article are solely those of the authors and do not necessarily represent those of their affiliated organizations, or those of the publisher, the editors and the reviewers. Any product that may be evaluated in this article, or claim that may be made by its manufacturer, is not guaranteed or endorsed by the publisher.

## Footnotes

^1^ https://osf.io/3hc7n/?view_only=49848537a6c543d7807020537d5da0b0
