# Supplementary material for: Children’s social networks in developmental psychology: A network approach to capture and describe early social environments
Source: Front Psychol. 2022 Oct 14;13:1009422. doi: 10.3389/fpsyg.2022.1009422 (PMC9614093; doi:10.3389/fpsyg.2022.1009422)
Supplement: Supplementary file 1 [file Data_Sheet_1.docx]

**Appendix A**

**The Child Social Network Questionnaire – How to run the Parent Interview**

The **Social Network Survey** has two parts. The first part is a parent interview where parents describe their child’s “typical week” of activities. This interview generates the people or ‘nodes’ in the child’s social network. The second part of the survey collects demographic information for each of the people mentioned. Below is a detailed guide on how to conduct the parent interview. These are prompts to use after you explain how the survey is going to work.

**Intro:** “Before I have you describe [CHILD’S NAME] typical week, can you tell me who are the people that live at home?”

- Questions you might get in response to this:
  - “They have an older sibling that doesn’t live at home anymore”
    - siblings should be included in the network
  - “We are a military family and their [mom/dad/step-mom/step-dad] is away right now”
    - That person should be included in the network. This is only true for Mom/Dad/step-mom/step-dad.
  - “My child lives in two different homes” – this happens when the child’s parents are not together. Depending on their response is how you proceed:
    - If the parent **can** tell you about their child’s experience in the other home – who lives there, who the child sees when they are with the other parent – you can proceed with the interview.
    - If the parent **cannot** tell you about their child’s experience in the other home or says something like “I couldn’t tell you” or “I only have them on weekends”, then you need to void the survey – you cannot be confident in this network data.
  - “Their grandma lives with us 6 weeks out of the year, but isn’t here right now.”
    - **Do not include** this person. The interview has to be conducted for the people the child sees **on a weekly basis**. The military family example is the only exception to this rule.

**Next prompt**: “Great, thanks! Now I want you to think about [CHILD’S] schedule Monday through Sunday – what does that look like for [HER/HIM] and what kinds of activities are they doing?”

- At this point, you will find out if the child is in daycare/preschool or not. If they are in daycare/preschool, this is the first things parents will tell you.
- **If they are in daycare/preschool:**
  - **“**How many teachers do they have at preschool?”
    - Questions that parents will ask in response to this question:
      - “Does the music/art/gym teacher count?”
        - If they see the music/art/gym teacher every week, then that person is included.
      - “They have 2 teachers and a teacher aide.”
        - The teacher aide is included.
      - “We go to a preschool where there is no set teachers – it is a lot of parents and adults and they change every week.”
        - Ask who were the adults that their child knows that they saw the previous week (this response is extremely rare – in over 300 interviews, this response happened once).
  - “Thinking about the kids in the class, is there anyone that stands out as a friend?
    - Questions that parents will ask in response to this question:
      - “They are friends with everyone.”
        - This is not true (and something we know from the Network literature – people are bad at representing their own networks). Do not disagree with the parent. Instead, ask, “Great, can you tell me their names?”. Parents will then produce a list of 1-5 friends on average. These children should be recorded as “friends” in the survey. See below about how this is a methodological choice in how the survey is conducted.
      - Kids in daycare/preschool will be its own node.
      - “I couldn’t tell you” / “You’re talking to the wrong parent”:
        - This is what I call the “doofy dad”. If you can get to the parents who can answer this, switch gears and interview them. If you cannot get the friends names, you have to exclude.
        - Importantly, this is different than “they have this new friend, but I can’t remember their name”. That is **okay**. You can say “just give us a name or I can leave them in here as “Friend 2” and you’ll know it is this person”.
- **If they are NOT in daycare/preschool:**
  - They will typically start describing their day with a primary caregiver (we read books, play with toys, etc). If they mention a person that wasn’t someone who lives at home (“they go to their Grandma’s house and see their cousin”) **make sure you include those people**.
  - If they go to a class every week, it will get mentioned here. See the next prompt for how extract those nodes.

**Next prompt**: “Great, thanks! Again, thinking about their schedule during the week and weekend, is there any other class or activity your child does every week? Either formal or informal - soccer or something like that?”

- Follow the same procedure as daycare/preschool. For each activity they list, you should ask:
  - “How many teachers/coaches do they have at [ACTIVITY]?”
    - Questions parents will have in response to this:
      - “They have 4 coaches, but they change every week”
        - Ask them who they saw the last time they were at the activity.
  - “Thinking about the kids in the class, is there anyone that stands out as a friend?
    - If they mention a friend from a different context “the friend Jason they have at preschool is also in basketball”, you already have that marked, so you do not need to mark it again.
    - “Kids from [ACTIVITY]” should be its own node

**Next prompt**: “Do you have extended family that you see on a WEEKLY basis?”

- Questions you will get in response to this:
  - “We see them once a month”
    - Do not include. They have to see them weekly or at the very least biweekly.
  - “We skype with Grandma every week”
    - If it is every week, then you should include this person.
  - “Yeah, we see them once a week or every other week.”
    - Include them.

**Next prompt**: “Great, so again thinking about [CHILD’S] typical week, is there anyone else that you think is worth mentioning?”

- Here is where I most often get “neighbors” and “family friends”. Parents will ask “Do you want the adults too?” IF they see the adults on a weekly basis, then yes!
- If this person they mention is a friend of their child, put the person in as a “Friend”. Put anyone else as the “Other” node.

**Appendix B**

**Demographic Form**

**Name:_____________________________________**

**Race (circle all that apply):**

African or African-American Asian or Asian-American

European or White-American Hispanic or Latino-American Native American

Biracial

Other:_______________________________

**Gender:**

Female Male Other:_______________________________

**Age:** _____________

How many/which languages does this person speak? Do they speak directly to the child in all these languages?

Please circle the categories that your child does with this person:

Eats meals with child.

Caretaking (changes clothes, bathes, etc.)

Play activities (plays with toys, watching TV, etc.)

Traveling outside the home (errands, grocery shopping, pick up siblings, etc.)

Outings (go to the park, museums, etc.)

Please write YES or NO to the following questions. There is space below if you feel the need to elaborate.

- - When your child is in distress, will they accept comfort from this person?
  - Does your child know this person’s name?
  - Do they talk about this person if the person is not present?
  - Will your child leave with this person (if this person came to your house and say “let’s go out and play”?)

**Appendix C**

**Museum Demographic Form**

**
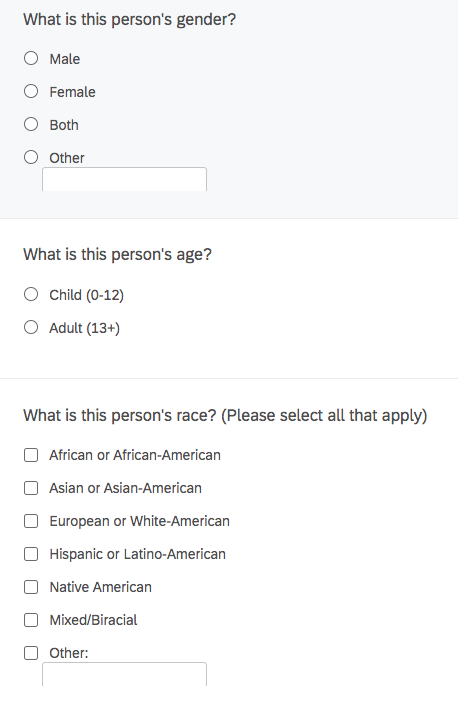
**

**
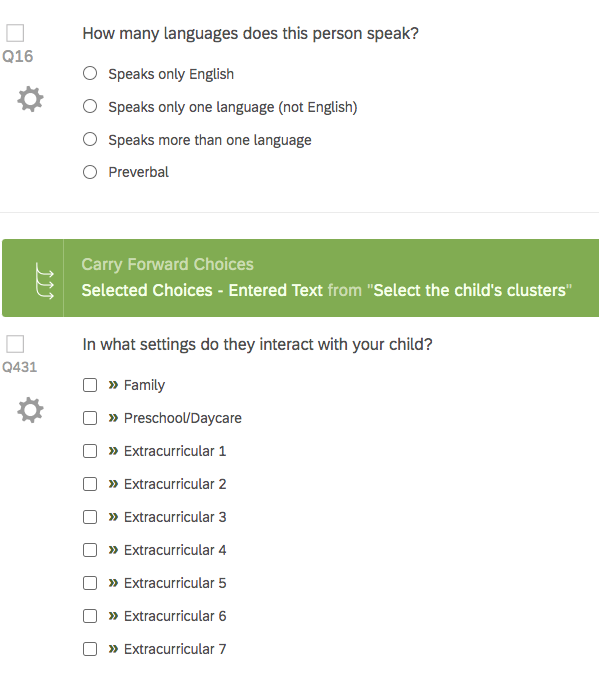
**
