# Supplementary material for: Children’s social networks in developmental psychology: A network approach to capture and describe early social environments
Source: Front Psychol. 2022 Oct 14;13:1009422. doi: 10.3389/fpsyg.2022.1009422 (PMC9614093; doi:10.3389/fpsyg.2022.1009422)
Supplement: Supplementary file 2 [file Data_Sheet_2.docx]

**Supplemental Materials**

**High and Low Intensity Relationships**

Below is a histogram of the intensity z-score for all 1232 social relationships. Relationships to the left of the red line were “low” intensity relationships and relationships above the red line were “high” intensity relationships.

**Figure 4**

*Histogram of The Relationship Intensity Z-Scores*


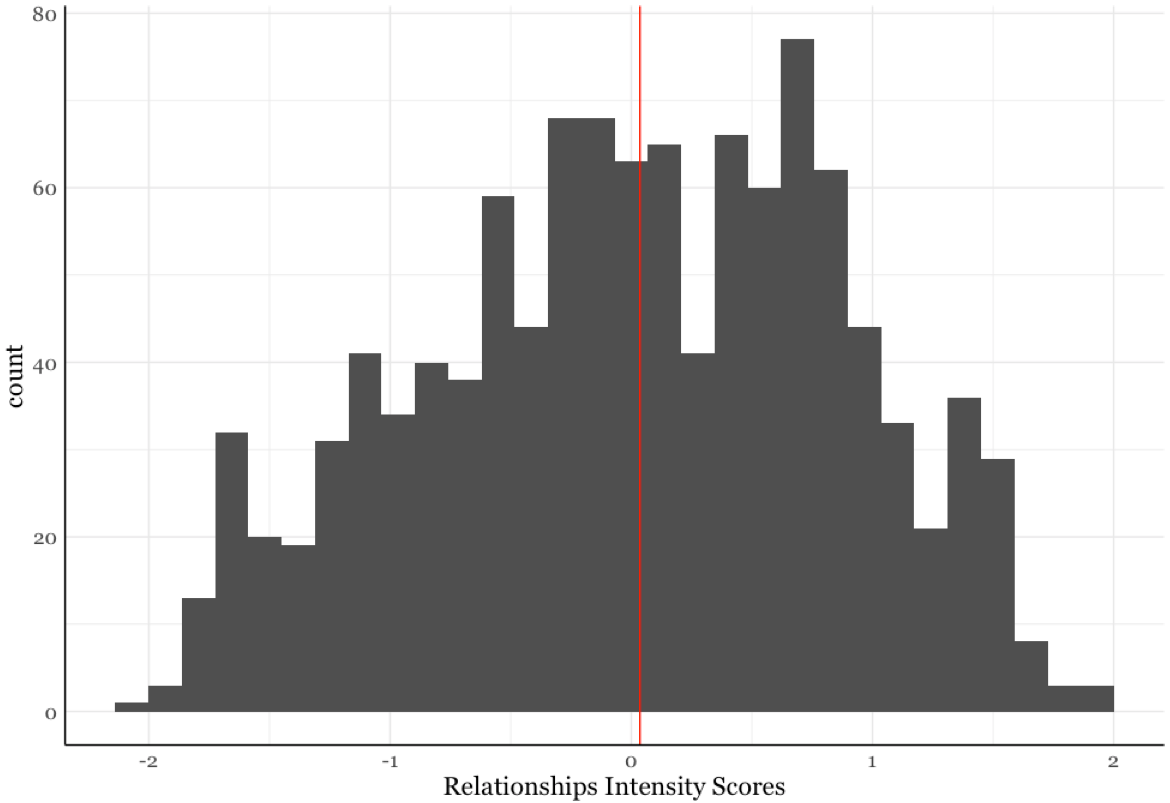


*Note*. The red line represents the median used to classify relationships as either “low” or “high” intense.

**Visual Representations of Diversity Measures**

**Entropy**

Entropy indicates the relative presence of different social categories among the alters in a network and is calculated as follows for a given probability vector of P(X): *H(X) =* - *∑ P(X) * log2(P(X))* (Drost, 2018). A score of 0 indicates that there is no diversity of categories; all the alters share the same attribute (e.g., all the alters are the same race). A higher entropy scores indicates a greater representation of different categories. See the pictures below for visual representation of Racial Entropy.

**
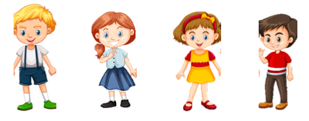
Figure 5**

*Example Network Racial Entropy Values*


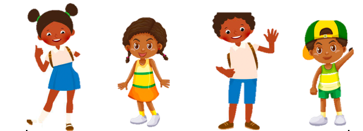
**
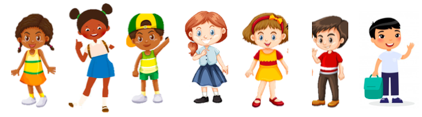
**

Network Racial Entropy = 0

Network Racial Entropy = 0.99

Network Racial Entropy = 0

*Note.* Above are examples networks and their corresponding Network Racial Entropy score. A score of 0 indicates that there is no racial diversity; all the people in the network are the same race.

**EI Index**

The EI Index is a measure of homophily the child shares with the network and is calculated as follows: *(Number of Different Alters – Number of Same Alters)/Network Size* (Krackhardt & Stern, 1988). The EI Index ranges from -1 to 1; a score of -1 indicates the entire network is the same as the child on some attribute and a score of 1 would indicate that the entire network is different from the child on some attribute (e.g., if a White child had a network where all the alters were White, they would get a score of -1). See the pictures below for visual representation of Racial EI Index for a White subject.

**Figure 6**

*Example Racial EI Index Values*

**
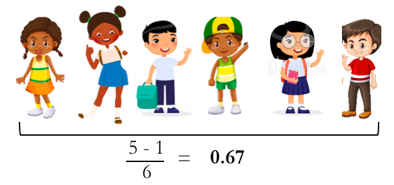

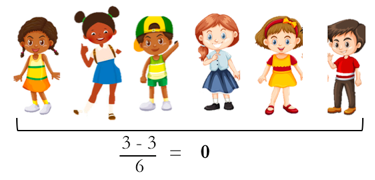

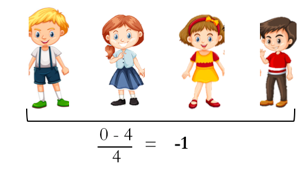

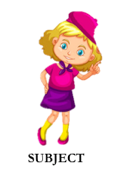
**

Example Network 2

Example Network 3

Example Network 1

*Note*. The pictures above show what the Racial EI Index would be if the child was White. A score of -1 means the entire network is the same race as the child.

**Network Size, Out-of-home-childcare, and Child Age**

In the sample, 167 subjects had out-of-home childcare either through school or daycare (*M_age_* = 37.8 months, *SD_age_* = 14.8 months) and 112 subjects (*M_age_* = 26.3 months, *SD_age_* = 11.8 months) did not have out-of-home childcare. This allowed us to explore how the network size changed as a function of childcare experience. For this sample, children who attended school or daycare were significantly older than children who did not attend school or daycare (*t*(269) = 7.2, *p* <0.001); therefore, age was included as a control variable in the subsequent analysis.

A linear regression was conducted to test the effects of Age and Out-of-home childcare on Network Size and the regression was significant (*R^2^* = 0.39, *F*(3, 275) = 61.3, *p* < 0.001; Figure 7). Consistent with the previous finding, there was a main effect of age (*ß* = 0.02, *p* < 0.001), but no main effect of Out-of-home childcare (*ß* = 0.10, *p* = 0.62), and no significant interaction (*ß* = 0.006, *p* = 0.28). Although it is true that children in out-of-home childcare had larger social networks than children without out-of-home childcare (*M_OutofHomeChildcare_* = 13 people (5), *M_NoOutofHomeChildcare_* = 9 people (5); *W* = 5031, *p* < 0.001), when controlling for the effect of Out-of-home childcare on Network Size, child age was the significant predictor.

Post-hoc, Bonferroni corrected correlations were performed to explore if the age trend is present for both children with and without out-of-home childcare. For both groups of children, there was a significant, positive correlation with age (Out-of-home childcare: *r* = 0.61, *p* < 0.001; No Out-of-home childcare: *r* = 0.41, *p* < 0.001). Regardless of childcare experience, as children got older their networks got larger. Notably, this relationship was weaker for children without out-of-home childcare.

**Figure 7**

*Network Size, Age, and Out-of-Home Childcare Experience*

*
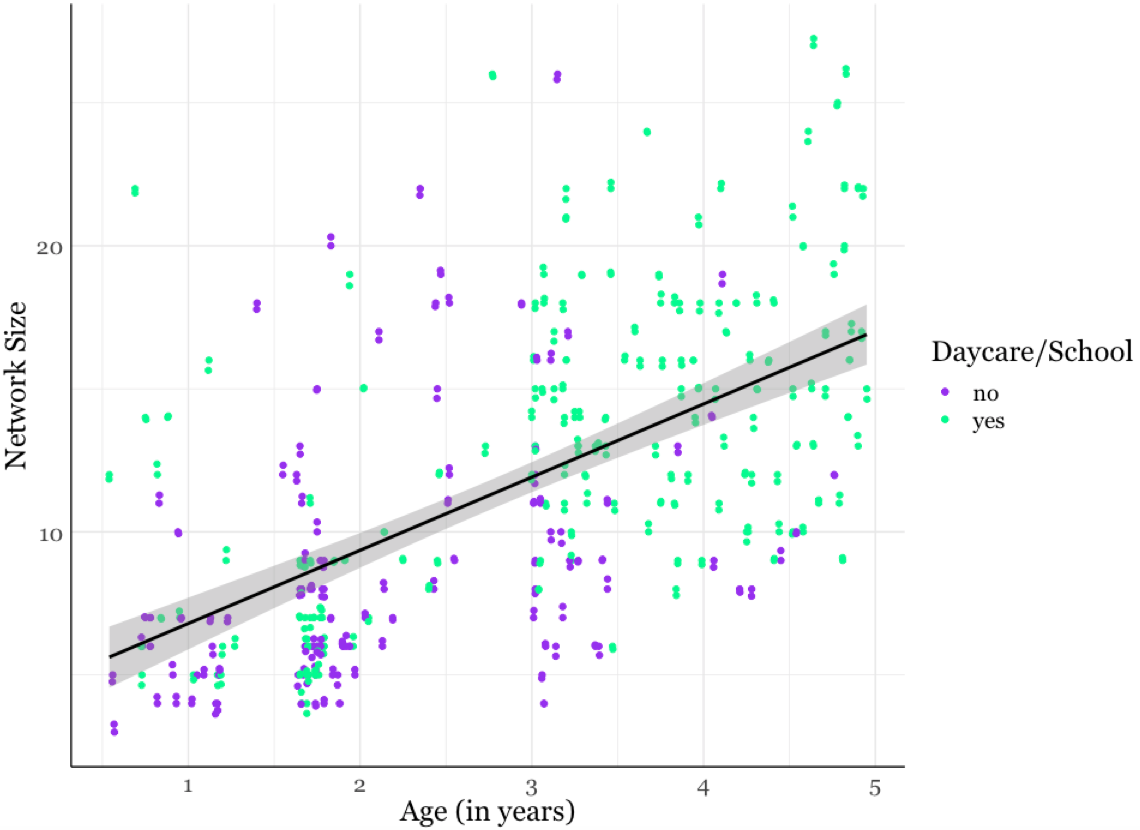
*

**Kin and Intensity Relationships**

Below is a table that presents the counts of kin, not kin, high intensity, and low intensity relationships for all 1232 social relationships.

**Table 4**

*2x2 Table Showing Kin and Intense Relationships*

|  | High Intense Relationships | | Low Intense Relationships | |
| --- | --- | --- | --- | --- |
|  | *n* | *% of total* | *n* | *% of total* |
| Kin Relationships | 471 | 42% | 212 | 19% |
|  |  |  |  |  |
| Not Kin Relationships | 90 | 8% | 349 | 31% |

**Network Variables and Child Age**

Below we present the scatterplots between the network variables and child age for completeness. The correlations are reported below, as well as in the manuscript.

**Figure 8**

*Proportion of High and Low Intense Relationships with Age*

*
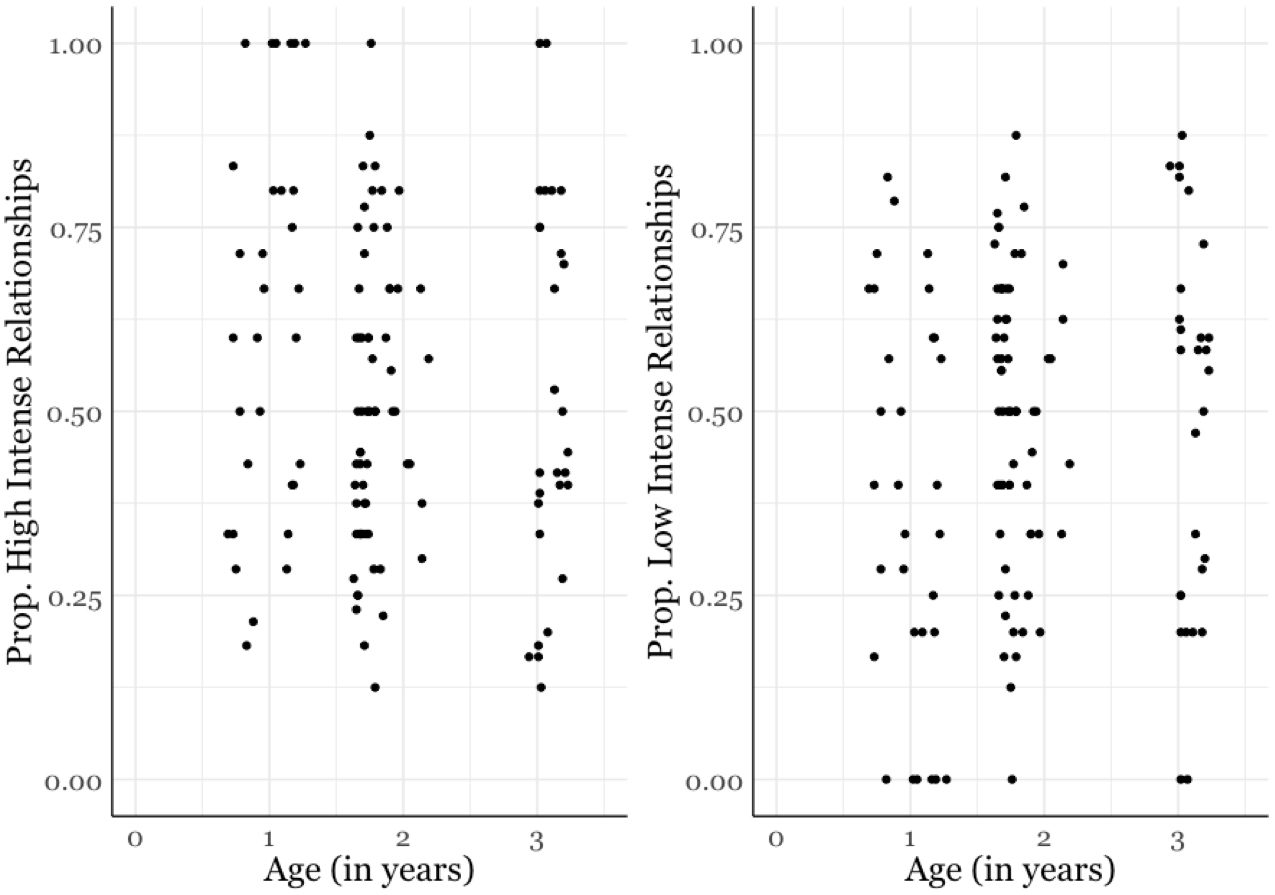
*

*Note*. There was no evidence that the proportion of high intense relationships (*rho* = -0.03, *p* = 0.72) or proportion of low intense relationships (*rho* = 0.03, *p* = 0.72) were correlated with age.

**Figure 9**

*Proportion of Kin Relationships with Age*

*
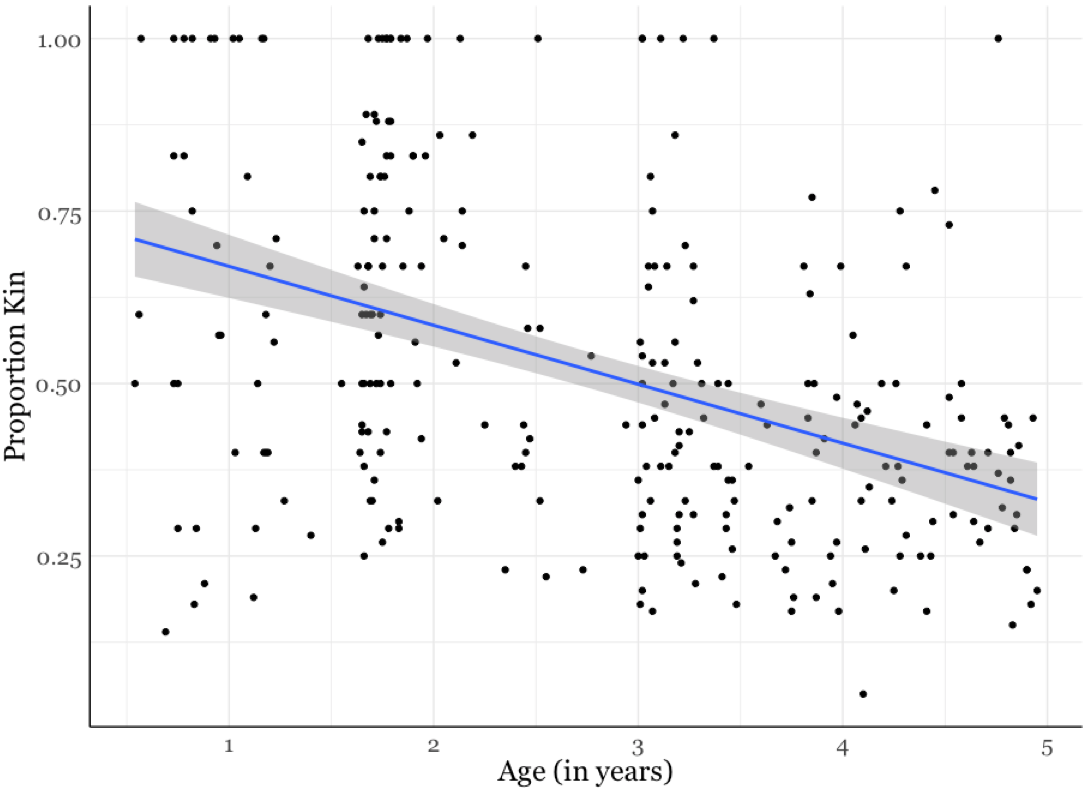
*

*Note.* Proportion of kin was correlated with child age (*rho* = -0.41, *p* < 0.001).

**Figure 10**

*Proportion of Adult Relationships with Age*

*
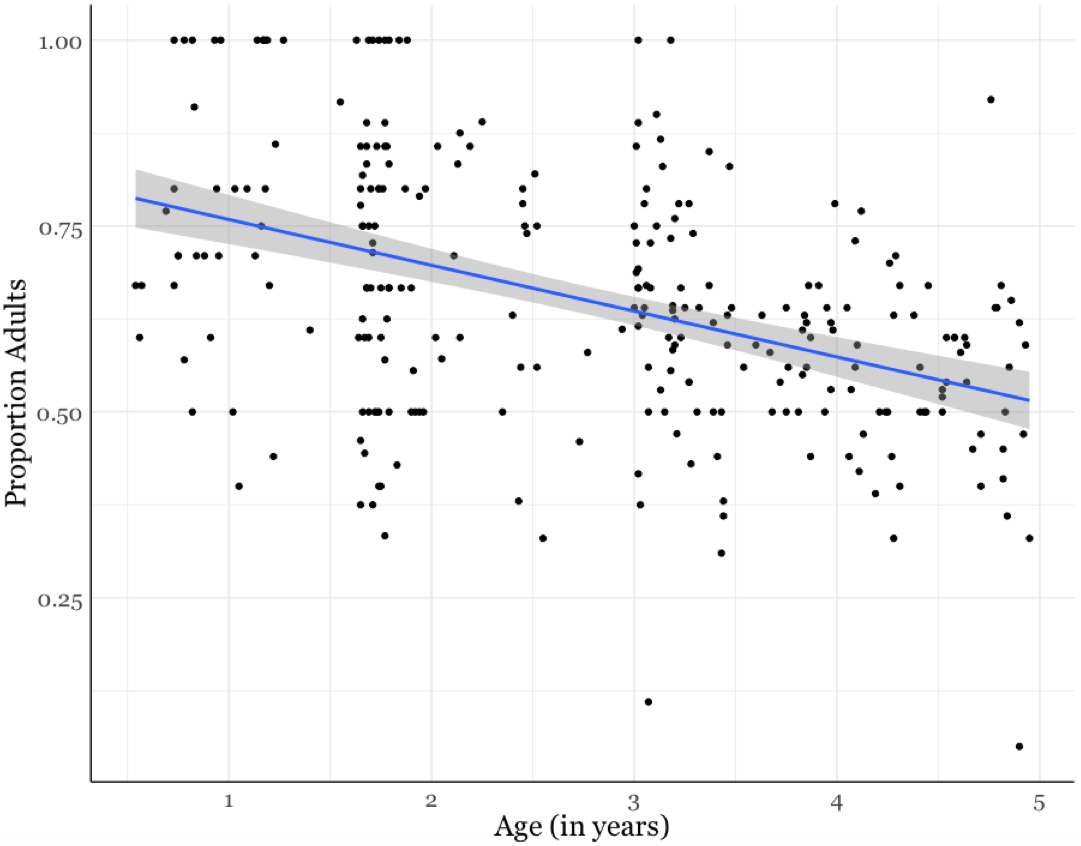
*

*Note.* Proportion of adult relationships was correlated with child age (*rho* = -0.42, *p* < 0.001).

**Figure 11**

*Network Racial Diversity and Age*

*
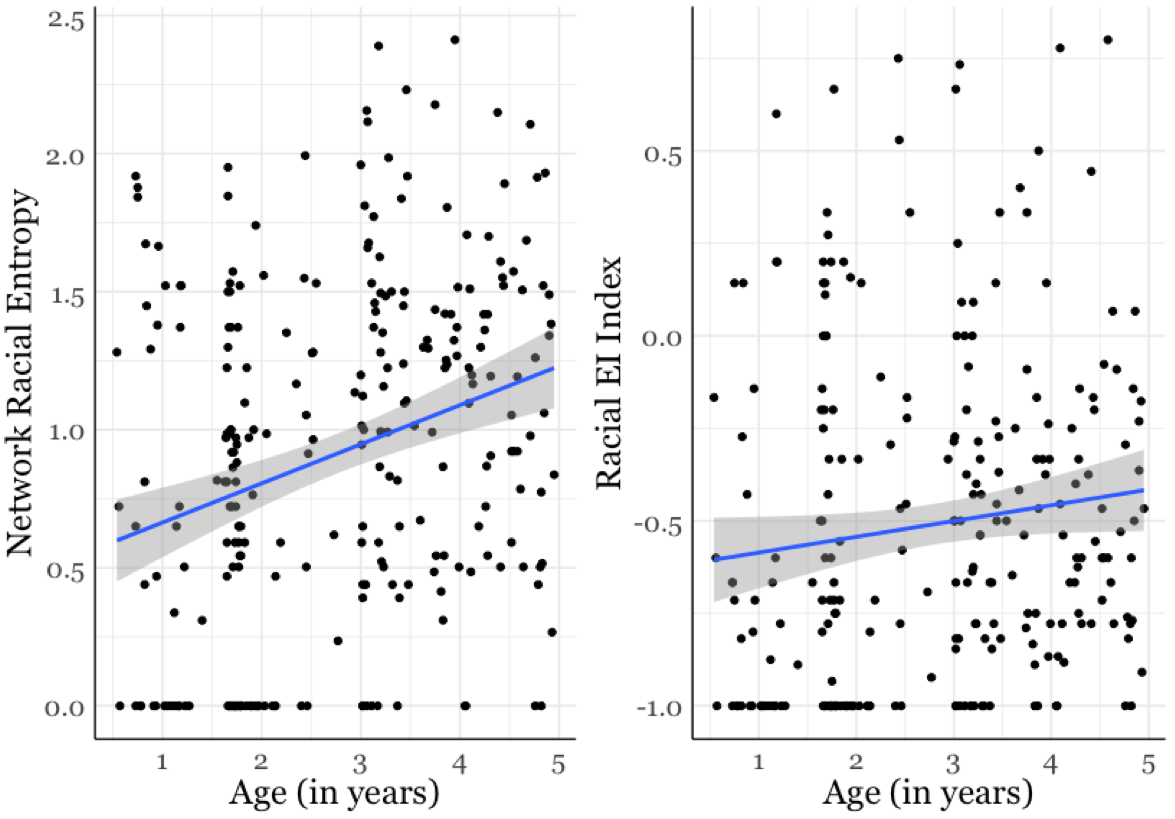
*

*Note*. There was a significant positive correlation with age for Network Racial Entropy (*rho* = 0.25, *p* <0.001) and for Network Racial EI Index and age (*rho* = 0.16, *p* = 0.01).

**Figure 12**

*Network Language Diversity and Age*

*
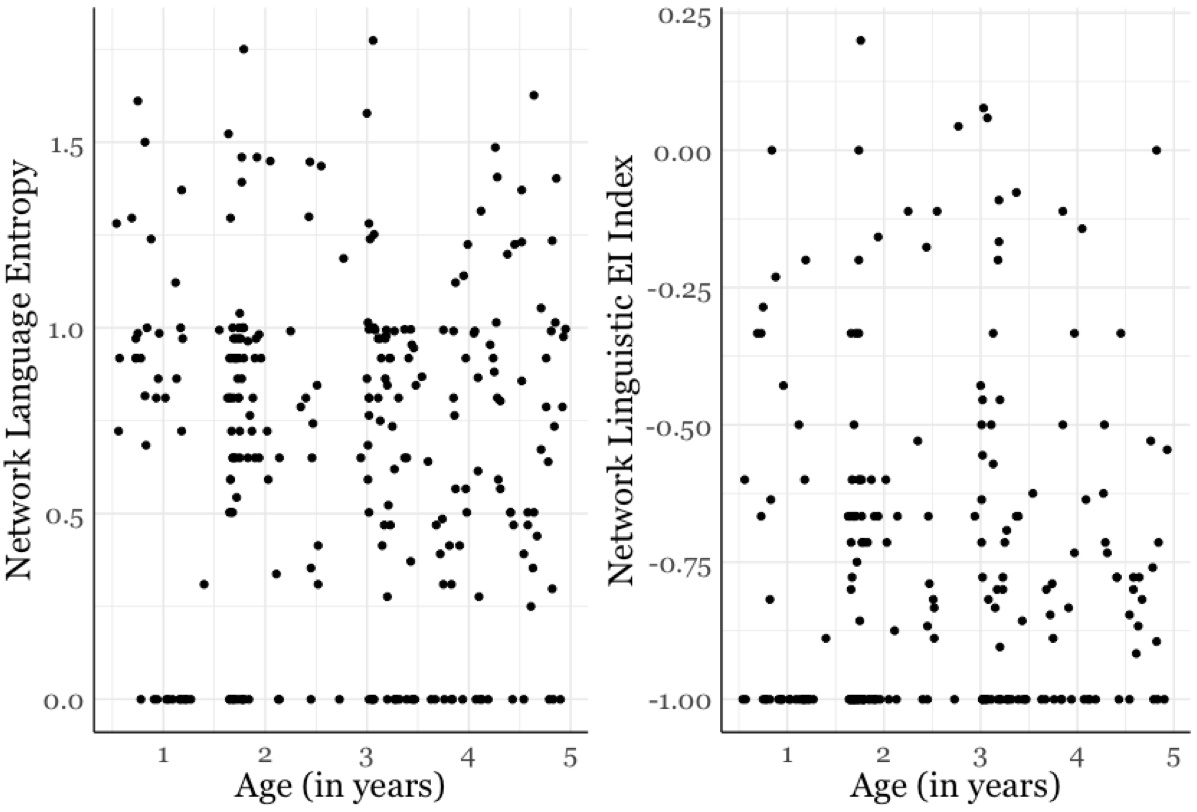
*

*Note*. There was no evidence that Network Language Entropy (*rho* = -0.07, *p* = 0.29) or Network Linguistic EI Index (*rho* = 0.09, *p* = 0.28) was correlated with child age.

**Figure 13**

*Density and Age*

*
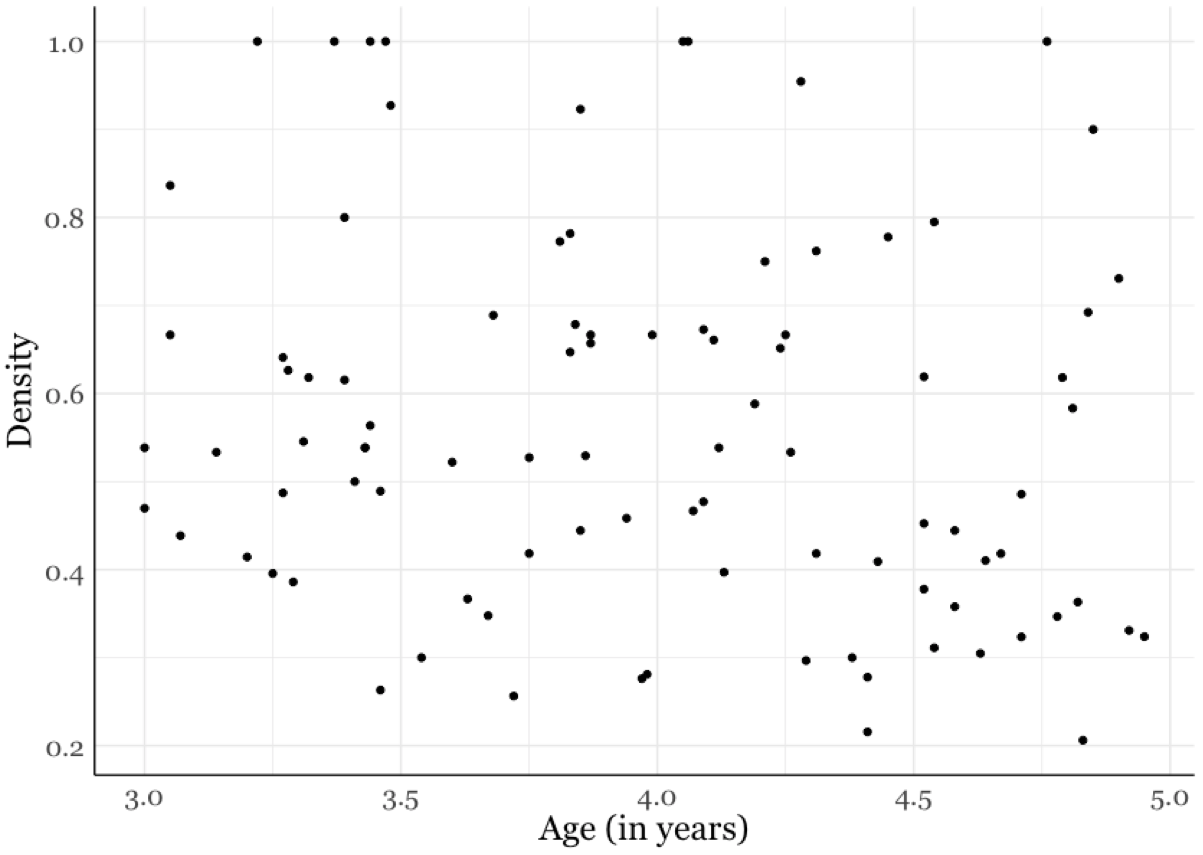
*

*Note*. There was no evidence that Density was related to child’s age (*rho* = -0.19, *p* = 0.12).

**Figure 14**

*Number of Components, Component Ratio and Age*


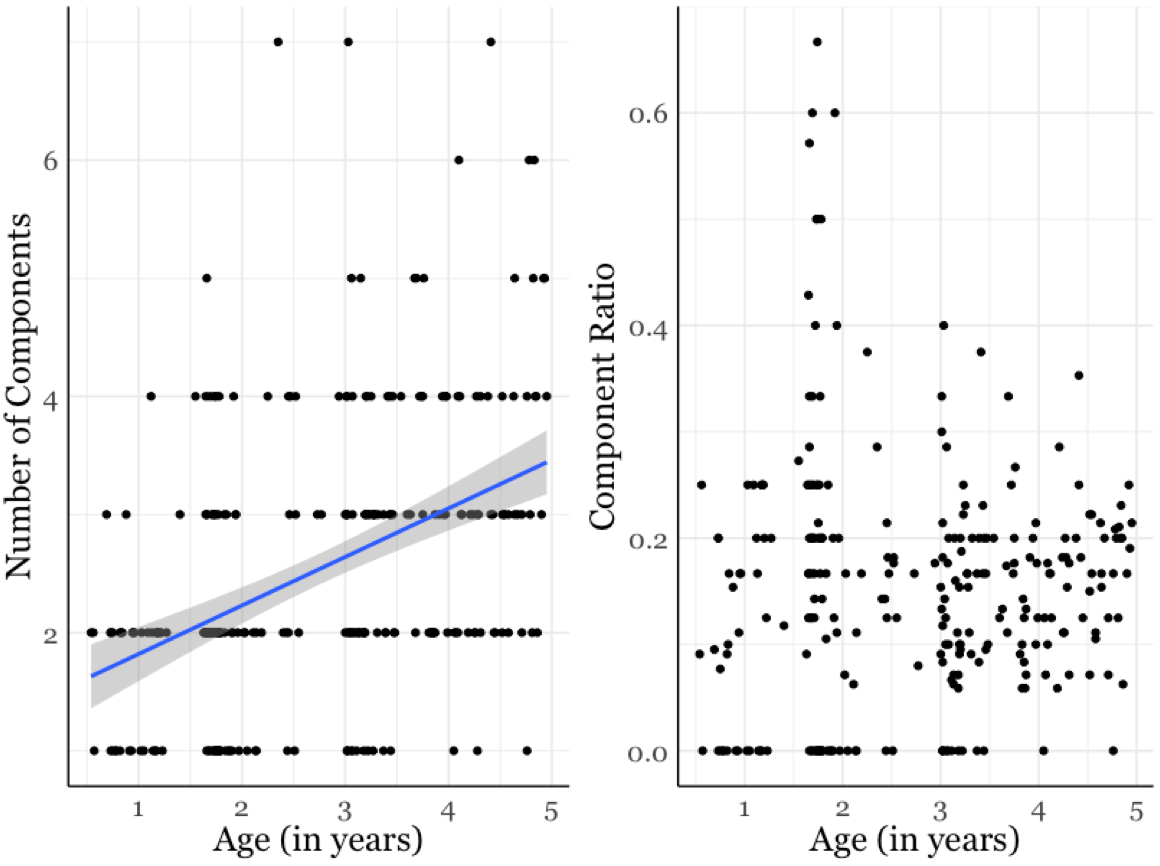


*Note*. The number of components was positively correlated with age – as children got older the number of components in their network increased (*rho* = 0.63, *p* < 0.001). The Component Ratio was not correlated with age; there was no evidence that the fragmentation of children’s networks varied with age (*rho* = 0.04, *p* = 0.59).

**Diversity in Children’s Social Networks**

**Entropy and EI Index**

Below are the scatterplots for both racial and linguistic entropy and EI Index. Both measures of diversity for both racial and linguistic diversity were highly correlated with each other.

**Figure 15**

*Network Racial Entropy and Racial EI Index*

*
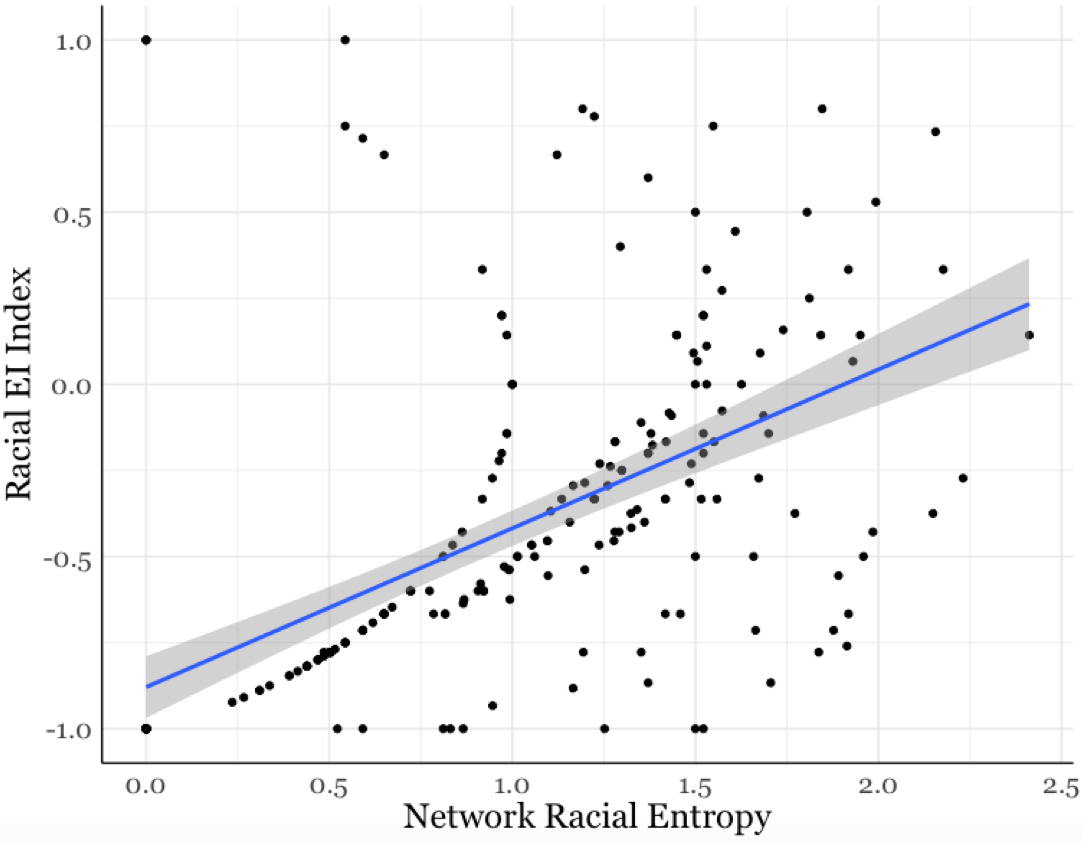
*

*Note.* The two measures of network racial diversity were highly correlated with each other (*rho* = 0.72, *p* < 0.001).

**Figure 16**

*Network Language Entropy and Linguistic EI Index*

*
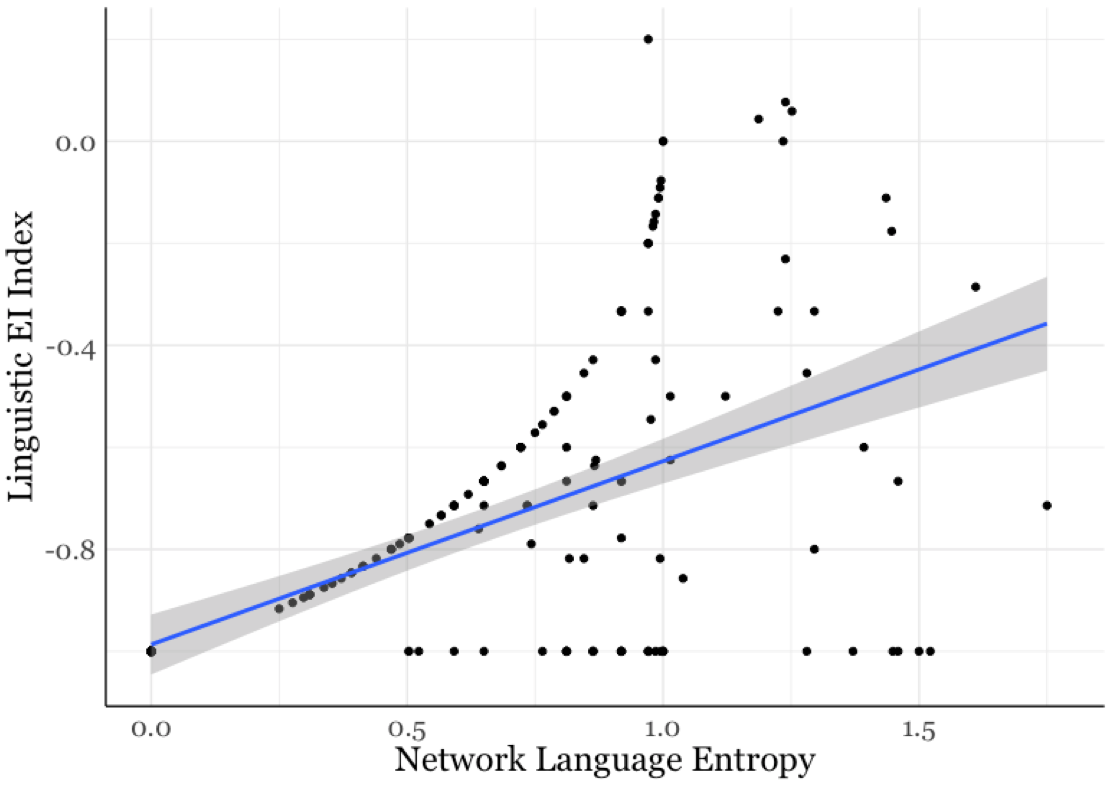
*

*Note.* The two measures of network linguistic diversity were highly correlated with each other (*rho* = 0.50, *p* < 0.001).

**Network Racial Diversity and Network Structure**

Below are examples of an Integrated Network and a Segregated Network.

**Figure 17**

*Examples of Integrated and Segregated Networks*

*
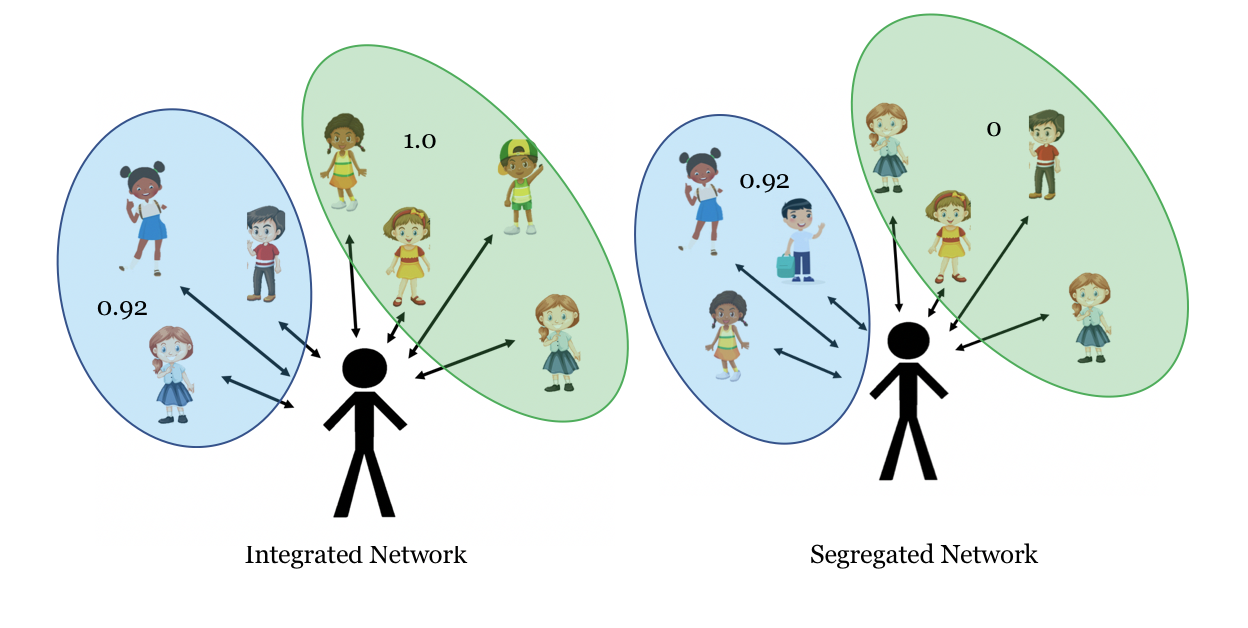
*

*Note.* The Integrated Network has 2 components that have non-zero entropy in each component – there are different racial groups represented in each component. The Segregated Network shows that 50% of the components have 0 racial entropy.
